# Urokinase Plasminogen Activator Inhibits HIV Virion Release from Macrophage-Differentiated Chronically Infected Cells via Activation of RhoA and PKCε

**DOI:** 10.1371/journal.pone.0023674

**Published:** 2011-08-17

**Authors:** Francesca Graziano, Chiara Elia, Carlo Laudanna, Guido Poli, Massimo Alfano

**Affiliations:** 1 AIDS Immunophatogenesis Unit, Division of Immunology, Transplantation and Infectious Diseases, San Raffaele Scientific Institute, Milan, Italy; 2 Vita-Salute San Raffaele University, School of Medicine, Milan, Italy; 3 Department of Pathology & Diagnostic, Faculty of Medicine and Surgery, Verona, Italy; University Hospital Zurich, Switzerland

## Abstract

**Background:**

HIV replication in mononuclear phagocytes is a multi-step process regulated by viral and cellular proteins with the peculiar feature of virion budding and accumulation in intra-cytoplasmic vesicles. Interaction of urokinase-type plasminogen activator (uPA) with its cell surface receptor (uPAR) has been shown to favor virion accumulation in such sub-cellular compartment in primary monocyte-derived macrophages and chronically infected promonocytic U1 cells differentiated into macrophage-like cells by stimulation with phorbol myristate acetate (PMA). By adopting this latter model system, we have here investigated which intracellular signaling pathways were triggered by uPA/uPAR interaction leading the redirection of virion accumulation in intra-cytoplasmic vesicles.

**Results:**

uPA induced activation of RhoA, PKCδ and PKCε in PMA-differentiated U1 cells. In the same conditions, RhoA, PKCδ and PKCε modulated uPA-induced cell adhesion and polarization, whereas only RhoA and PKCε were also responsible for the redirection of virions in intracellular vesicles. Distribution of G and F actin revealed that uPA reorganized the cytoskeleton in both adherent and polarized cells. The role of G and F actin isoforms was unveiled by the use of cytochalasin D, a cell-permeable fungal toxin that prevents F actin polymerization. Receptor-independent cytoskeleton remodeling by Cytochalasin D resulted in cell adhesion, polarization and intracellular accumulation of HIV virions similar to the effects gained with uPA.

**Conclusions:**

These findings illustrate the potential contribution of the uPA/uPAR system in the generation and/or maintenance of intra-cytoplasmic vesicles that actively accumulate virions, thus sustaining the presence of HIV reservoirs of macrophage origin. In addition, our observations also provide evidences that pathways controlling cytoskeleton remodeling and activation of PKCε bear relevance for the design of new antiviral strategies aimed at interfering with the partitioning of virion budding between intra-cytoplasmic vesicles and plasma membrane in infected human macrophages.

## Introduction

Urokinase-type plasminogen activator (uPA) is a serine protease that activates plasminogen to plasmin [1] and is synthesized as an inactive precursor (pro-uPA) that undergoes proteolytic activation. Both pro-uPA and uPA bind to a specific glycosyl-phospatidyl-inositol (GPI)-anchored receptor, uPAR, on the cell surface [2] and are expressed by inflammatory cells including neutrophils, mononuclear phagocytes (MP), and activated T lymphocytes [2] for which cells they play important roles in cell activation, adhesion and migration. In addition to focusing the proteolytic activity of uPA on the surface of the cells, extracellular uPAR acts as a functional receptor of uPA-dependent signaling [3], inducing cell adhesion, migration, and proliferation in different cell types independently of its catalytic component [4], [5].

Of particular relevance is the fact that the uPA system seems to represent an overlapping mechanism involved in both tumor and HIV disease progression [6]. The full-length soluble form of uPAR (suPAR) is predictive of negative clinical outcome in different diseases including cancer [7] and HIV/AIDS. In this latter condition, suPAR represents a novel prognostic indicator which was shown to be independent from and as indicative as low numbers of circulating CD4^+^ T cells or high viremia levels [8]. Furthermore, suPAR correlated with the state of immune activation of HIV-infected individuals, as well as with the lipid and glucose metabolism [9]. Of note, higher levels of suPAR were demonstrated not only in the plasma/serum of HIV^+^ individuals [10], [11], but also in the central spinal fluid (CSF) of neurologically compromised HIV^+^ individuals [12], [13] and correlated with CSF viral load [13]. Since we have previously reported that uPA inhibits the release of HIV virions from monocytic cells by inducing their accumulation into intracellular vesicles [14], independently of its enzymatic activity but through binding to uPAR followed by vitronectin dependent adhesion [15] these observations suggest that the increased levels of suPAR may work as a soluble receptor complexing and preventing uPA from exerting an anti-HIV activity *in vivo*.

Since uPAR is GPI-anchored receptor with no intracellular domain it requires the interaction with signaling-competent transmembrane proteins for triggering signal transduction [16]. To date, the most well characterized uPAR partners include integrins [3], the G-protein coupled receptor formyl peptide receptor ligand 1 (FPRL1) [3], and gp130, the signal transducing subunit of the IL-6 R family [17]. uPAR and integrins act as a single functional unit in several cells. Of interest is the fact that monocytes from uPAR-deficient mice fail to adhere onto fibrinogen and to migrate upon thioglycollate stimulation [18] in spite of the expression of the signaling-competent integrin CD11b-CD18 (Mac-1). In this regard, we have recently reported that uPA increased the adhesion to the substrate of both primary human monocyte-derived macrophages (MDM) and of chronically infected promonocytic U1 cells differentiated to macrophage-like cells by phorbol myristate acetate (PMA). Adhesion to the substrate was responsible for uPA-mediated inhibition of HIV expression in these infected cells and was mediated by vitronectin (VN) binding to the uPA/uPAR complex [15]. The ability of the uPA/uPAR/VN complex to redirect virions into intra-cytoplasmic vesicles was independent of the catalytic activity of uPA but was induced by the amino-terminal fragment (ATF) of uPA, competent for intracellular signaling [14], [15], [19], [20]. The general mechanism of uPA-mediated inhibition of HIV replication in macrophages is related to the induction of an increased accumulation of virions budding into intra-cytoplasmic vacuolar compartments of debated nature, a peculiar feature of infected MP not observed in CD4^+^ T lymphocytes [21]. This morphological feature of MP has suggested the model of infected macrophages as “Trojan horses” of HIV infection contributing to the generation/maintenance of viral reservoirs in anatomical sites such as the CNS [22]. In addition to other stimuli, including interferon-γ (IFN-γ) [23], CCL2 [24], and Mac-1 ligands [14], the uPA/uPAR/VN system might thus represent a mechanism leading to intracellular entrapment of virions into the vesicles.

In this study, we have investigated the nature of the intracellular signaling triggered by uPA/uPAR interaction responsible for the intracellular accumulation of HIV virions. In particular, we have focused our attention on the small GTPase RhoA and PKC isoforms, previously reported to mediate cell motility, chemotaxis, adhesion, migration and stress fiber formation [25], activities that can be mediated by uPA/uPAR interaction independently of uPA catalytic activity [4], [5]. In this regard, we have previously demonstrated that uPA induces similar HIV virion morphogenetic features in PMA-differentiated adherent U1 cells [19], [26] and in primary MDM either infected *in vitro* [19] or differentiated *ex-vivo* from monocytes of infected individuals [15]. The present study has been performed in the convenient model of PMA-differentiated U1 cells permitting a broader range of experimental conditions than primary MDM obtained from different donors.

## Materials and Methods

### Reagents

LPS-free (bacterial endotoxin <2×10^−5^ EU/IU corresponding to <10^−10^ EU/mg) human uPA (M.W. of 52 kDa) was provided by Dr. Jack Henkin (Abbot Laboratories, IL, USA) and Andrew P. Mazar (Chemistry of Life Processes Institute, Evanston, IL), and was used at 10 nM based on our previous studies [14]. This concentration reflects uPA binding affinity for uPAR (0.1–1 nM) [27] and its levels determined in pathological conditions [28], including serum levels in HIV-1^+^ individuals [29]. Hoechst-33342, DNase I Alexa Fluor 488, phalloidin Alexa Fluor 633 were from Molecular Probes (Eugene, OR). Triton X-100, crystal violet, rabbit anti-goat FITC antibodies (Ab) and phorbol-12, myristate-13, acetate (PMA) were purchased from Sigma Chemical Corp. (St. Louis, MO), monoclonal Ab (mAb) against different PKC isoforms were obtained from BD Transduction Laboratories (B-9320 Erembodegem, Belgium), and polyclonal Ab against the phosphorylated form of PKCε and PKCδ from Santa Cruz Biotechnology (Santa Cruz, CA). PMA was resuspended in ethanol at 1 mM and used at 10 nM. Cytochalasin D was from Calbiochem (Merck KGaA, Darmstadt, Germany) and was resuspended in DMSO and used at 10 µM, based on preliminary experiments identifying non-toxic concentrations for U1 cells, defined as [^3^H]-thymidine uptake and vital cell counts after Trypan blue staining. Penetratin-1 (P1) and P1 fusion peptide containing RhoA/23–40 (P1/23–40), RhoA/92–119 (P1/92–119) and PKC myristoylated pseudosubstrate peptides were dissolved in DMSO at 10 mM and were used at the final concentration of 50 µM [30]. Paraformaldehyde (PFA) was purchased from Merck. Round coverslips were from Zeus (Zeus Scientific, Branchburg, NJ), whereas microscope glass slides were obtained from BDH (Poole, UK). Mowiol 4–88 was bought from Calbiochem. Experiments were performed in either standard tissue culture plates (Falcon, BD Biosciences, Bedford, MA) or in Teflon-coated ultra-low adhesion (ULA) plates to rule in/out the contribution of cell adhesion to the investigated hypothesis.

### Chronically HIV-infected U1 cell line

The promonocytic U1 cell line was originally obtained from a population of U937 cells surviving the cytopathic effect of acute CXCR4-dependent HIV-1_LAI/IIIB_ infection; each U1 cell contains 2 copies of integrated proviruses [26]. No virus production is detected by conventional reverse transcriptase (RT) activity assay or p24 Gag antigen ELISA in cultures of unstimulated U1 cells, but virus expression at levels comparable to those achieved at peak of acute virus infection of parental U937 cells or primary MDM and T cell blasts is rapidly induced by stimulation of U1 cells with PMA or pro-inflammatory cytokines [26]. U1 cells (2×10^5^ cells/ml, unless otherwise indicated, in RPMI 1640 containing 10% of heat-inactivated FCS, 1% pen/strep and 1% glutammine) were incubated with uPA (10 nM), PMA (10 nM) or PMA+uPA in the presence or absence of the above mentioned peptides and reagents. All experiments were performed with U1 cells kept in culture medium containing 10% of FCS because serum starvation (observed at FCS concentrations <2%) prevents uPA-induced adhesion and inhibition of HIV expression, likely consequent to the reduced amounts of VN in the culture medium [15]. Thus, we decide to keep the 10% of serum in our culture medium in order to allow the proper formation of the uPA/uPAR/VN system, and likely the generation of unbiased intracellular signal(s).

### Supernatant- vs. cell-associated virus

HIV-1 virion release in culture supernatants was monitored by determination of Mg^++^-dependent RT activity. Since 99% of the RT enzyme is virion-associated, this assay is a faithful indication of the production of new progeny virions [31]. In order to quantify the levels of cell-associated RT activity, cells were washed twice with culture medium at room temperature and subjected to 5 consecutive cycles of cell freezing and thawing [14].

### U1 cell proliferation and adhesion assay

Cell proliferation and adhesion were measured after 48 h of stimulation in triplicate cultures in 96 flat-bottomed well tissue culture plates, as reported [14], [15].

### Optical microscopy

Pictures of cell cultures in the different experimental conditions were acquired with an optical phase contrast Leica DM IL microscope and analyzed by FireCam software (Leica, Deerfield, IL). Pictures of suspended cells or cell clustering were directly taken in the original culture well. Pictures of adherent cells were taken after removal of culture supernatants (i.e., including floating cells, such as present in PMA-stimulated U1 cells, representing about 60% of cells and segregated into the clusters), 2 washes with warm culture medium, 1 wash with 4% PFA in PBS, and 20 min fixation in PFA/PBS at room temperature.

### Quantification of polarized cells

U1 cells were washed and resuspended in culture medium without serum at concentration of 5×10^5^ cells/ml, and incubated with Cell Tracker Red CMPTX (1 µM, Invitrogen) for 30 min at 37°C, 5% CO_2_. Cells were then washed and resuspended at 25×10^3^ cells/ml in standard medium supplemented of 10% FCS and left untreated or incubated with RhoA and PKCs inhibitors for 45 min at 37°C, 5% CO_2_ before stimulation with PMA in the presence or absence of uPA. After 48 h cells were washed with warm PBS and adherent cells were fixed with warm 2% PFA/PBS for 15 min at room temperature. Intracellular actin was then stained with phalloidin-FITC (8 µM, Sigma) in 1 ml of saponin buffer (0.1% saponin and 0.5% BSA in PBS) for 30 min at room temperature. Cells were then washed twice with warm PBS and stained with Hoechst 33342 trihydrochloride trihydrate (Invitrogen) at the concentration of 3.24 µM in PBS for 15 min at room temperature. Cells were washed twice with PBS and maintained in PBS. Approximately 250 random fields were automatically acquired by the IN Cell Analyzer 1000 (GE Healthcare, Fairfield, CT), using objective magnification of 40×. Analysis was performed with In Cell Investigator Software: polarized cells were identified using the “form factor”, a standard estimate of circularity that relates perimeter length to area. This measurement varies from 0 to 1, with 1 being a perfect circle. A threshold of 0.8 was fixed for this parameter and cells with a “form factor” lower than threshold classified as polarized cells. Cell polarization was based on the form factor as automatically calculated by the IN Cell Investigator Software (GE Healthcare); ratio below 0.8 was used to identify polarized cells, because was identified as the threshold discriminating between round and stretched cell shapes. Picture montage was performed by using the ImageJ software.

### Intracellular distribution of filamentous and globular actin

U1 cells were stimulated with the reported stimuli and plated on top of round coverslip. Two days later coverslips were washed twice with PBS and adherent cells fixed with 4% buffered PFA for 20 minutes at room temperature, washed twice with PBS cells and permeabilized by 0.1% Tryton X-100 in PBS for 5 min at room temperature. After two more washes with PBS cells were stained with Hoechst-33342 (3.24 µM in PBS), DNase I Alexa Fluor 488 (0.3 µM in PBS) and phalloidin Alexa Fluor 633 (0.165 µM in PBS) for 20 min at room temperature. After two more washes with PBS cells were mounted and sealed on microscope glass slide with Mowiol 4–88. Images were acquired by Leica TCS SP2 confocal microscope (Leica Microsystems CMS GmbH, Mannheim, Germany) using objective magnification 63×. Picture montage was performed by using the ImageJ software.

### Ultrastructural study

U1 cells were stimulated with PMA in the presence or absence of uPA and aliquots of 5×10^6^ cells were then washed in Phosphate Buffered Saline (PBS) and analyzed after 48 h by electron microscopy as previously described [14]. Briefly, U1 cells were washed twice in PBS, fixed in 4% (vol/vol) glutaraldehyde/2% (wt/vol) PFA/cachodilate (0.12 M pH: 7.4) and postfixed in 1% (vol/vol) OsO4 (0.12 M) cachodilate buffer. Cells were dehydrated in graded ethanol, washed in propylene oxide and infiltrated for 12 h in a 1∶1 mixture of propylene oxyde∶epoxydic resin (Epon). Cells then were embedded in Epon and polymerized for 24 h at 60°C. Slides were cut with ultramicrotome (Ultracut Uct, Leica), stained with uranyl acetate and lead citrate, and metallized.

### Protein kinase C (PKC) translocation and activation

U1 cells were washed and aliquots of 5×10^6^ cells were resuspended in 12.5 ml of RPMI 10% FCS and either left unstimulated or were stimulated with PMA in the presence or absence of uPA at the indicated time points. U1 cells were then centrifuged at 1,200 rpm for 10 min at 4°C, the culture medium was removed and the cells were resuspended in 300 µl of cold homogenization buffer [Hepes/K (20 mM pH:7.6), EDTA/K (5 mM pH:8), EGTA/K (5 mM pH:7.6), DTT (5 mM), Na3VO4 (1 mM), NaF (2 mM)] and protease inhibitor cocktail (1∶1,000 vol/vol; Sigma). Cells were homogenized by 5 consecutive passages trough a 22G needle mounted onto a 1 ml syringe. The cell homogenates (250 µl) were transferred into ultracentrifuge tubes (Beckman Coulter, Fullerton, CA) and spun at 100,000 g for 60 min at 4°C (at 50,000 rpm in ultra TL-100 with the TLA-100.3 rotor or at 42,000 rpm with the TLA-55 rotor, Beckman). The supernatants containing cytosolic proteins were then collected, whereas the pellets were resuspended in 120 µl of the homogenization buffer supplemented of 1% TritonX-100, centrifuged at 100,000 g for 30 min at 4°C (Beckman) and the supernatants containing membrane proteins were collected. Finally, 10 µg of either cytosolic or membrane-associated proteins were loaded on 10% SDS-PAGE and analyzed by Western blotting using mAb directed against specific PKC isoforms α, δ, ε.

Cytofluorimetric analysis of phoshorylated PKC**ε** and PKCδ was performed on cells that were washed with cold PBS, fixed with 2% PFA/PBS for 15 min at room temperature, permeabilized with saponin buffer in the presence of the specific Ab for 30 min at room temperature, followed by one wash with saponin buffer and further 30 min of incubation with the secondary anti-goat FITC Ab. An average of 15,000 cells per condition was acquired using a FACScan (Becton Dickinson, Franklin Lakes, NJ) flow cytometry apparatus and analyzed by the CellQuest software (Becton Dickinson).

### Determination of RhoA activation

The amount of the active form of RhoA (GTP-bound RhoA) was assessed using the G-LISA RhoA Activation Assay Kit (Absorbance Based; Cytoskeleton, Inc., Denver, CO) according to the manufacturer's protocol. Cell (5×10^6^) were washed and resuspended in culture medium (1 ml) in the presence or absence of stimuli for the indicated time points and 10 µg of protein were used for the assay.

### Statistics

Each experiment was design to analyze cell adhesion, clustering and polarization as well as HIV expression in the same experimental set up. All experimental conditions were tested in duplicate wells. The results are reported as mean ± standard deviation (SD). Multivariate analyses were conducted by one-way ANOVA and by the Tukey post-test. Differences were considered significant if p<0.05.

## Results

### The intracellular accumulation of virions induced by uPA in PMA-differentiated U1 cells is mediated by activation of small GTPase RhoA and PKCε, but not PKCδ

**Figure 1:**
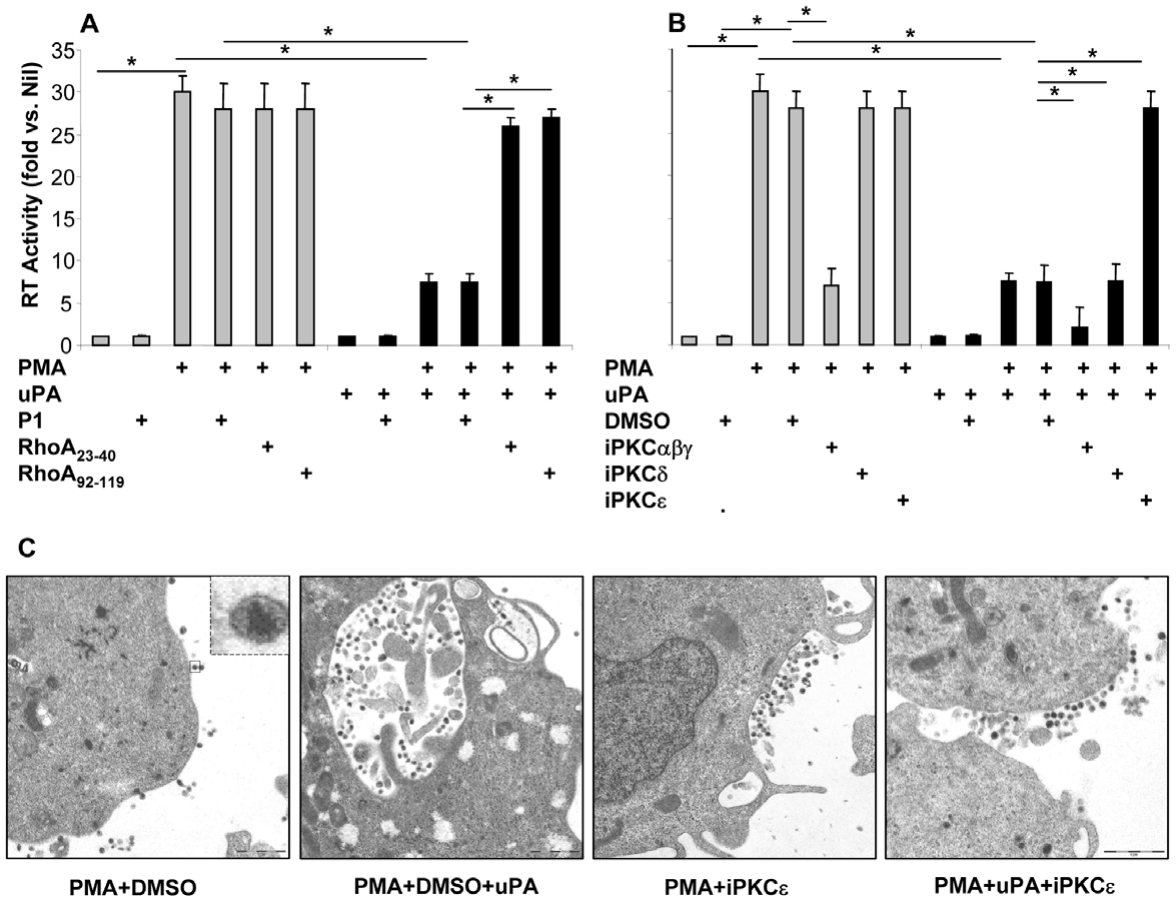
The anti-HIV activity of uPA is mediated by RhoA and PKCε, but not by PKCδ. U1 cells were pre-incubated for 45–60 min at 37°C with RhoA Trojan peptide (50 µM) (**A**) or myristoylated peptides specific for different PKC isoforms (50 µM) (**B**) and then stimulated with PMA (10 nM) in the presence or absence of uPA (10 nM); culture supernatants were analyzed 48 h later for the levels of virus expression (mean±SD of 11 independent experiments). Nil represents the negative background RT activity of unstimulated U1 cells (typically, 120±50 cpm/µl). P1; penetratin was used at 50 µM as negative control of Trojan peptides. DMSO is the vehicle in which PKC pseudosubstrate is resuspended, and here used as control. *p<0.05. (**C**) Cells were stimulated in the conditions specified above and analyzed by electron microscopy 48 h after stimulation; more extensive selection of pictures is represented in the Supplementary Figures S1 and S2. iPKC; inhibitor of the specified PKC isoforms.

We have previously reported that the cell adhesion–dependent antiviral signal triggered by uPA binding to uPAR requires β1 and β2 integrin chains [14]. We therefore investigated whether the small GTPase RhoA was mediating the intracellular accumulation of HIV virions observed upon uPA treatment of U1 cells stimulated with PMA since RhoA is known to influence cell adhesion by promoting integrin activation [32]. HIV expression from U1 cells was induced by PMA stimulation (Figure 1A and 1B), while uPA inhibited more than 50% of virus expression by promoting virion accumulation in intracellular vesicles (Figure 1C), as previously reported [14], [20].

The Trojan peptides P1/23–40 and P1/92–119 were previously shown to specifically target the small GTPase RhoA [30] thus inhibiting the transition from low to intermediate and to high affinity state and/or the lateral mobility of the β_2_ integrin leukocyte functional antigen-1 (LFA-1, also known as CD11a/CD18), respectively. RhoA trojan peptides did not influence HIV expression in unstimulated U1 cells (data not shown) or in cells stimulated only with PMA, while they abrogated the anti-HIV activity of uPA (Figure 1A). Penetratin1 (P1), the common fusogenic component of Trojan peptides, was used as control [30].

Since protein kinase C (PKC) represents a downstream effector of RhoA [30], [33], we next investigated whether activation of PKC isoforms was involved in uPA-dependent intracellular accumulation of virions in U1 cell upon PMA stimulation. Isoform-selective myristoilated pseudosubstrates peptides or control scrambled peptides were applied to U1 cells, in the presence or absence of PMA and uPA. As expected, blockade of Ca^++^-dependent PKCαβγ isoforms interfered with PMA-induced virus expression in the absence of uPA, consistently with previous reports on the role of PKC activation driving HIV expression [34]. Of interest, the anti-HIV effect of uPA in PMA-stimulated U1 cells was reversed exclusively by inhibition of PKCε, but not by PKCδ (Figure 1B). Ultrastructural analysis was performed on cells treated with PKC**ε** pseudosubstrate (Figure 1C and S1), showing that inhibition of PKCε activation prevented both the formation of cytoplasmic vesicles as well as virion accumulation into such compartment (Table 1). In contrast, PKCε pseudosubstrate did not show any effect in U1 cells stimulated with interleukin-6 (IL-6) (Figure S2), a cytokine triggering HIV expression in U1 cells without inducing cell adhesion and, therefore, insensitive to the inhibitory effects of uPA [15].

**Table 1:** PKCε and virion partitioning in stimulated U1 cells.

Stimuli	p.m.-associated virions	i.c. virions	% virions pm/ic	# vesicles per cell	# cells with vesicles/total
**PMA**	74	0	100	1	1/10
**PMA+iPKCε**	199	3	98	2	2/10
**PMA+uPA**	58	79	42	>10	9/10
**PMA+uPA+iPKCε**	165	5	97	1.5	5/10
**IL-6**	46	0	100	0	0/10
**IL-6+iPKCε**	105	0	100	0	0/10

U1 cells were pretreated with PKCε pseudosubstrate and stimulated for 48 h with the indicated stimuli, and analyzed by transmission electron microscopy as previously reported [14]. Unstimulated U1 cells did not show evidence of either virion production or cell vacuolization (not shown). p.m.: plasma membrane; i.c.: intracellular; iPKCε: inhibitor of the isoform PKCε.

All above inhibitors were used at concentrations that provide the maximal biological effect in the absence of evident cellular toxicity, as determined by cell proliferation (measured in terms of overnight uptake of ^3^H-thymidine) and cell viability (by Trypan-blue dye exclusion) [19] after 3 days of stimulation. A PKCζ pseudosubstrate was also tested, but resulted in either toxic effects on the cells or in no effect on cell adhesion when used at non-toxic concentrations (data not shown).

### uPA-induced cell adhesion and cell polarization is mediated by small GTPase RhoA, PKCδ and PKCε

**Figure 2:**
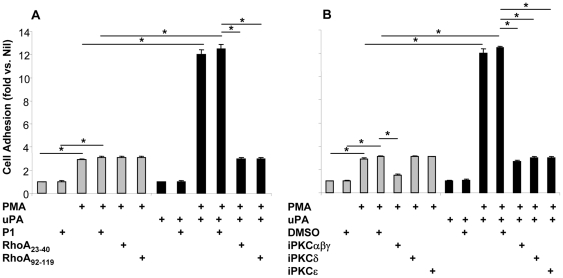
uPA induces adhesion of PMA-differentiated U1 cells via activation of small GTPase RhoA, PKCδ and PKCε. U1 cells were pre-incubated for 45–60 min at 37°C with RhoA Trojan peptide (**A**) or myristoylated peptides specific for different PKC isoforms (**B**) and then stimulated with PMA in the presence or absence of uPA; cells were analyzed 48 h later for their capacity to adhere to the plastic substrate. uPA increased the adhesion of PMA-stimulated U1 cells and this effect was reversed by inhibition of either RhoA or different PKC isoforms. Results are shown as mean ± SD of 11 independent experiments. Nil indicates unstimulated U1 cells, showing a mean net absorbance value (subtracted of the background value of an empty well) of 0.07±0.012. *p<0.05.

PMA stimulation of myelo-monocytic cell lines, including promonocytic U937 and U1 cell lines, induce their activation and differentiation along the macrophage lineage [26]. Such a process is associated with the transition from cell proliferation in suspension to a drastic decrease of the proliferative capacity and acquisition of cell adhesion to the substrate. As we have previously demonstrated in primary MDM [15], PMA-induced U1 cell adhesion to the substrate was enhanced in the presence of uPA (Figure 2A). Although both RhoA Trojan peptides failed to affect PMA-induced cell adhesion; however, they fully prevented the enhancement of adhesion to the substrate induced by uPA (Figure 2A).

Next, we investigated whether activation of PKC isoforms was involved in uPA-dependent increase of U1 cell adhesion following PMA stimulation. In this regard, different studies have reported that both PKCα and ζ regulate, concurrently with RhoA, β_1_ and β_2_ integrin-dependent adhesion resulting in cell spreading and lateral mobility of LFA-1, respectively [30], [35]. As in the case of HIV expression, blockade of Ca^++^-dependentαβγ PKC isoforms also interfered with PMA-induced cell adhesion (Figure 2B). Furthermore, it has been reported that both uPA and ATF can lead to formation of diacylglycerol (DAG) that, in turn, can induce the activation of PKC δ and ε isoforms [36] associated with promotion of cell adhesion and migration of human epithelial cells [37], [38]. Consistently, pharmacological targeting of PKC δ and ε isoforms did not influence PMA-dependent cell adhesion but fully prevented its enhancement induced by uPA (Figure 2B).

Concomitantly with the enhancement of cell adhesion, uPA inhibited homotypic cell aggregation, as reported [15]. PMA induced formation of both adherent cells and of cell aggregates that remained in suspension (Figure S2). Cell aggregation was abolished and all cells were adherent to the substrate when co-stimulated with PMA and uPA (Figure S2A). Trojan peptides targeting the small GTPase RhoA did not modify PMA-induced cell clustering but fully reversed the inhibitory effect of uPA (Figure S3A). PMA-induced homotypic clustering of U1 cells was not affected by the PKC pseudosubstrates tested (Figure S3A–B). In addition, interference with PKC δ or ε, but not with the αβγ isoforms, reversed the uPA-dependent inhibition of cell aggregation (Figure S3B).

**Figure 3:**
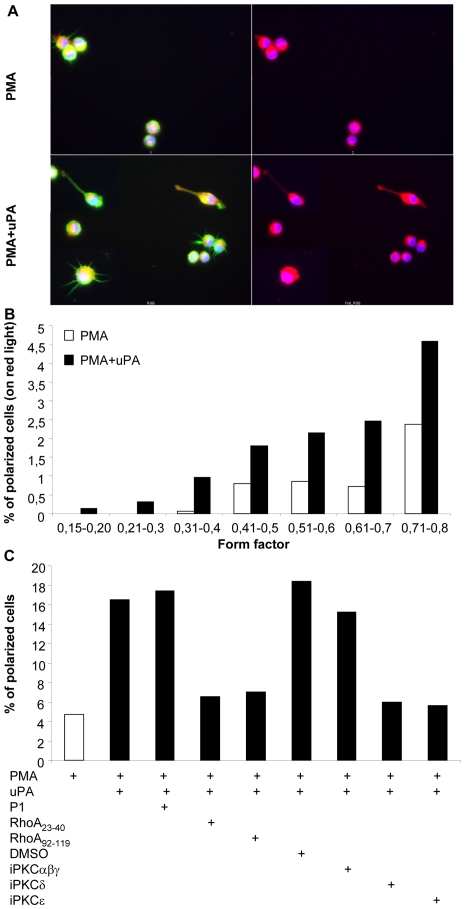
uPA induces polarization of PMA-differentiated U1 cells. U1 cells were loaded with cell tracker (red color) and then stimulated with PMA in the presence or absence of uPA. Adherent cells were stained 2 days later for F-actin (phalloidin-FITC, green color) and nuclei (blue color). A total of 1,142 and 2,245 adherent cells were counted after stimulation with either PMA alone or in the presence of uPA, respectively. (**A**) uPA induces polarization of U1 cells with formation of uropods, not observed in control cells stimulated only with PMA. (**B**) Quantification and distribution of polarized cells, as defined by form factor <0.8. (**C**) Inhibition of anti-RhoA or PKC δ and ε isoforms reverted uPA-induced cell polarization: this analysis is based on the distribution of the cell tracker (right panels, red light) since staining of F-actin (left panels, green light) revealed the presence of filopodia, cell surface projections highly enriched in F-actin required for the formation of focal adhesion sites [73] that may produce false-positive results.

In parallel to the induction of cell adhesion (and to the prevention of cell aggregation) uPA induced a morphological polarization of U1 cells, as previously observed in the case of neutrophils [39], vascular smooth muscle and epithelial cells stimulated by uPA or ATF [37]. Polarized cells were characterized by the formation of classical uropods (Figure 3A and Figure S4) that were only present in adherent cells. This change in cell shape was still observed 48 h after stimulation. This finding is in accordance to previous findings showing that binding of uPA to uPAR on the surface of U937 cells is long-lasting, with ca. 80% of input uPA bound to uPAR after 4 h [40]. Furthermore, uPA proteolytic activity in the monocytic cell line THP-1 has been shown to last for 40 h or longer [40]. Uropods formation was indeed not observed when cells were cultivated in ULA plates (data not shown), characterized by low binding of serum proteins and lack of cell adhesion [15]. Unbiased quantification of the number of polarized cells was performed on randomly selected fields acquired by InCell analyzer (Figure 3A), with the cells being analyzed for their width/length ratio by the InCell Investigator software excluding from the analysis cells partially out of the field (Figure S5). As observed by phase contrast microscopy (Figure S4), the InCell Investigator software quantified that 4.7% and 15% of the adherent U1 cells were indeed polarized after PMA or PMA+uPA, respectively (Figure 3B). Furthermore, PMA-stimulated U1 cells were characterized by the absence of uropods, whereas co-stimulation with PMA and uPA induced a more evident cell polarized characterized by a lower width/length ratios and induced the formation of uropods (Figure 3B). Inhibition of RhoA or PKCδ or PKCε activities did not affect PMA-induced cell polarization (data not shown) but abolished the enhancement of cell polarization induced by uPA to values similar to those observed in PMA-stimulated cells (Figure 3C). In contrast, RhoA Trojan peptides did not show any effect in U1 cells stimulated with interleukin-6 (data not shown), a cytokine triggering HIV expression in U1 cells without inducing a macrophage-like phenotype, therefore insensitive to the inhibitory effects of uPA [15].

### uPA induces actin rearrangement in PMA stimulated U1 cells

**Figure 4:**
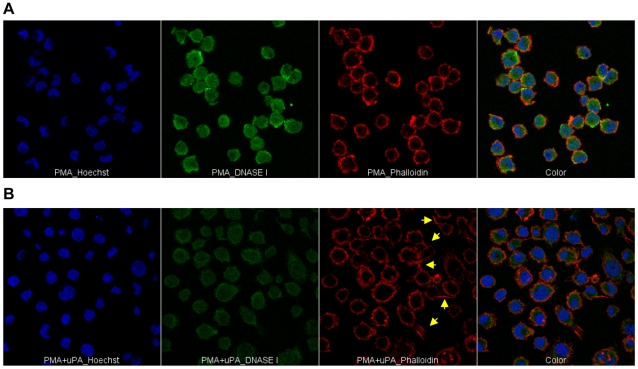
Different actin distribution in uPA polarized cells. U1 cells were stimulated with PMA in the presence or absence of uPA and actin distribution in adherent cells was visualized by confocal microscopy after 48 h of culture. Hoechst-33342, DNase I (Alexa Fluor 488, green) and phalloidin (Alexa Fluor 633, red) were used to discriminate nuclei, globular (G actin) and filamentous (F actin) isoforms, respectively. Images were acquired with 63× magnification by Leica TCS SP2 confocal microscope (Figure S6) and electronically zoomed 3 times. Image J software was used to perform montage of the three colors. Yellow arrows indicate polarized cells. One experiment out of three independently performed with similar results is shown.

Since both cell adhesion and polarization are dependent on cytoskeleton re-organization, intracellular distribution of globular (G) and filamentous (F) actin was investigated. Two days after PMA or PMA+uPA stimulation adherent U1 cells were stained with probes specific for G and F actin, such as DNaseI and phalloidin, respectively. In PMA-stimulated U1 cells G actin was homogenously distributed, with F actin was localized below the cellular membrane (Figure 4A) when U1 cells were stimulated by PMA in the presence of uPA G actin was homogenously distributed (Figure 4B), although levels of intensity were decreased (Figure 4B and Figure S7A). However, uPA did not alter actin expression [14] indicating that decreased levels of G actin intensity are consequent of G to F switch, likely due to an increased volume of uPA stimulated cells (Figure 3A and Figure S7A). Indeed, distribution of F actin in PMA+uPA stimulated cells overlapped to that observed in PMA stimulated cells, such as below the cellular membrane, but also in tails present only in polarized cells (Figure 4B).

### uPA induces RhoA activation in PMA stimulated U1 cells

**Figure 5:**
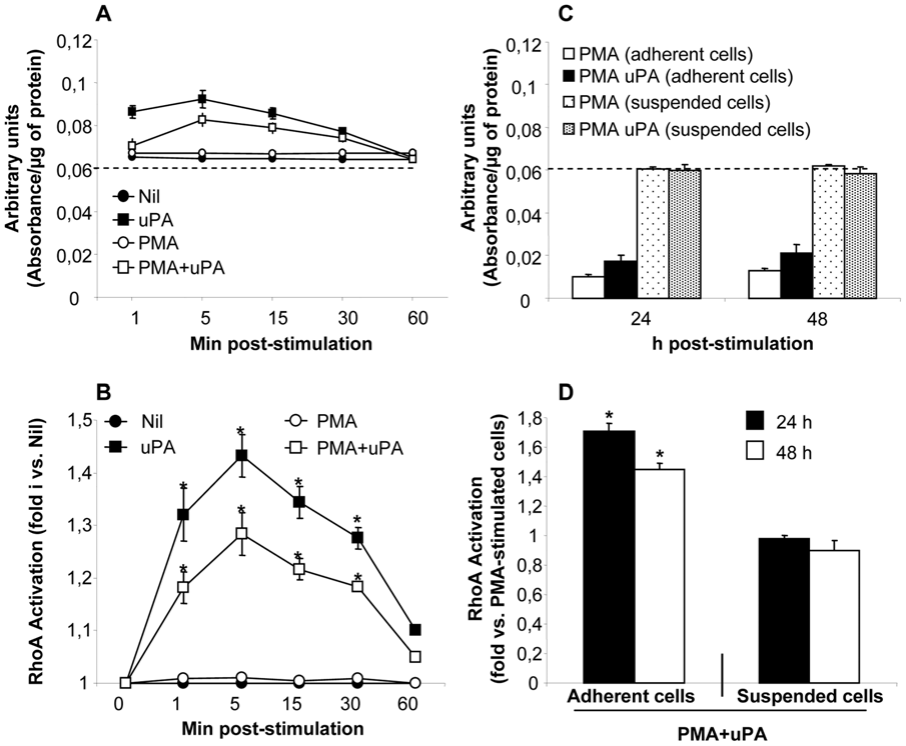
uPA induces two waves of RhoA activation in PMA-activated U1 cells. Quick RhoA activation in U1 cells upon uPA stimulation: absorbance indicating RhoA activation was measured by the G-LISA kit assay and was normalized per µg of protein (**A**) and results of 3 independent experiments reported ad fold induction (mean ± STD) vs. unstimulated cells (**B**). Delayed uPA-induced activation of RhoA is adhesion-dependent: RhoA activation was measured in PMA or PMA+uPA stimulated U1 cultivated in culture plates (adherent cells) or in Teflon-coated plates not allowing adhesion (suspended cells) (**C**), and results of 3 independent experiments reported ad fold induction (mean ± STD) vs. PMA stimulated cells. The dotted line in panels A and C indicate the levels of RhoA activation observed in unstimulated cells (panel **A**) and in cells maintained in suspension in ULA plates after stimulation for 24–48 h with PMA (**C**). Calpeptin (2 U/ml for 15 min) was used as positive control and induced 2±0.1 fold of RhoA activation vs. Nil (not shown).

In order to assess the activated form of RhoA we measured the GTP-bound form of RhoA by G-LISA. Unstimulated (Nil) and PMA-stimulated cells showed equal levels of activated RhoA (Figure 5A), confirming independent studies indicating that PMA failed to activate RhoA in fibroblats [41]. In contrast, uPA induced RhoA activation in both unstimulated and PMA-stimulated U1 cells (Figure 5A and 5B). RhoA activation reached a peak of activation as soon as 5 min after uPA stimulation, returning to basal levels after 60 min (Figure 5A and 5B). It should be underscored that U1 cells at these early time points are still completely in suspension, and that the same findings were observed in ultra-low adhesion (Teflon-coated) plates (data not shown).

The levels of RhoA activation were also analyzed after 24 and 48 h of culture. When both unstimulated and PMA-stimulated cells were maintained in suspension in Teflon-coated plates the levels of RhoA activation were constant over time (from 5 min to 48 h, Figure 5A and 5C, dashed lines), whereas it decreased by ∼6 folds after 24–48 h in standard plates allowing cell adhesion (Figure 5C and 5D), as described [32]. In contrast, uPA stimulation caused an increased in RhoA activation exclusively in cells that were allowed to adhere, but not in cells cultivated in Teflon-coated plates, as observed after 24–48 h of culture (Figure 5C and 5D). Thus, a second wave of RhoA activation, likely consequent to integrin outside-in signaling, is generated and maintained upon uPA-induced cell adhesion.

### uPA induces activation of PKC ε and δ isoforms

**Figure 6:**
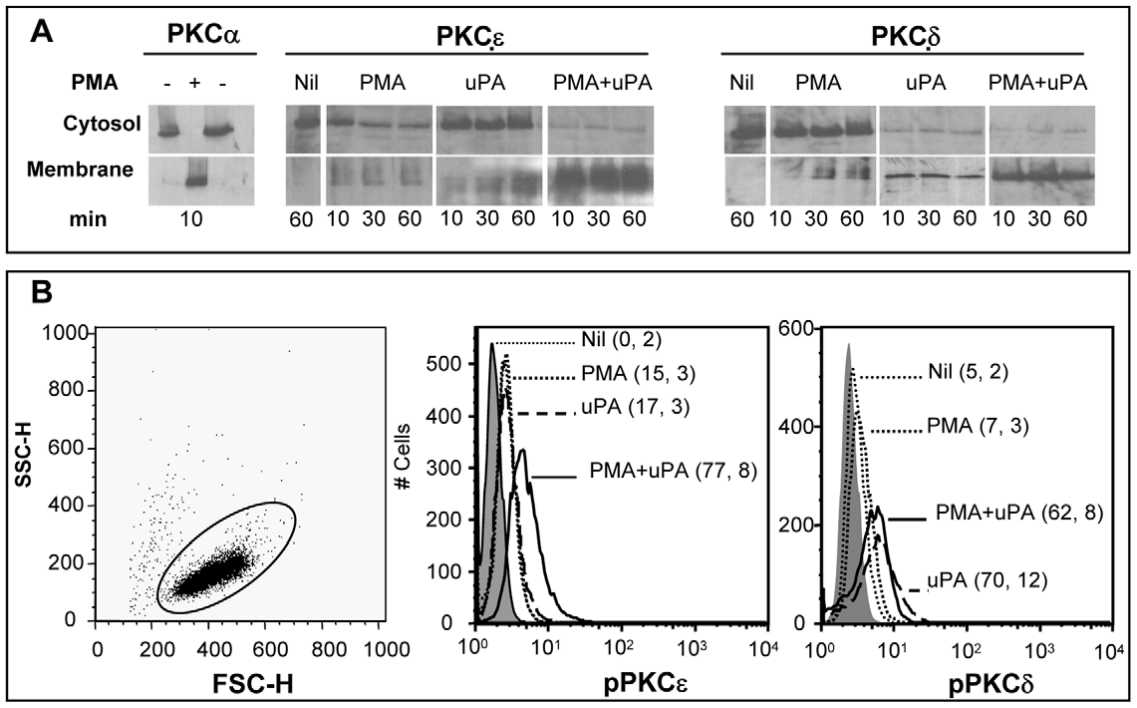
uPA induces activation of PKCδ and ε in PMA-stimulated U1 cells. PKC activation was measured by two independent assays, such as cytosol-membrane translocation of PKC and measurement of PKC isoforms phosphorylation. Cells were left either unstimulated or were incubated with PMA in the presence or absence of uPA. (**A**) Membrane and cytosolic fractions of U1 cells were analyzed for the presence of PKC isoforms at the indicated time points; membrane and cytosolic extracts were run independently but in a single gel per fraction. (**B**) Cells were analyzed by cytofluorimetric means for the presence of phosphorylated PKCε and PCKδ 30 min after stimulation (numbers in parentheses represent the % of positive cells and geometric means of fluorescence, respectively). The results shown in A and B were obtained from a single experiment representative of 5 independently performed.

We measured the activation of PKC isoforms in terms of their translocation from the cytoplasm to the membrane [42] (Figure 6A) as well as of the presence of phosphorylated isoforms (Figure 6B). As expected, PMA induced the complete translocation of PKCα from the cytosol to the plasma membrane as tested 10 min after cell stimulation (Figure 6A), an event that was unaffected by uPA (data not shown). PKCε was detected exclusively in the cytosolic fraction of unstimulated U1 cells while PMA poorly induced its translocation to the plasma membrane (Figure 6A). Stimulation of U1 cells with uPA alone induced a time-dependent PKCε translocation to the membrane (Figure 6A). When cells were co-stimulated with PMA and uPA, however, a synergistic effect was observed on the translocation of this PKC isoform from the cytosol to the membrane that was already maximal 10 min after stimulation and remained elevated for at least an additional 50 min.

In comparison to PKCε PKCδ showed faster kinetics of translocation from the cytosol to the plasma membrane that was already maximal 10 min after stimulation with uPA in the absence of PMA (Figure 6A). Like PKCε, also for PKCδ the strongest activation was induced by simultaneous co-stimulation of U1 cells with PMA and uPA. This pattern is likely explained by the observation that PMA has been shown to enhance uPAR affinity for uPA, either directly or indirectly affected by PKC [43].

Since membrane fractionation does not allow isolation of plasma membrane from total membranes, a second method for analyzing PKC activation was used, and intracellular levels of activated isoforms was performed by cytofluorimetric analysis. U1 cells stained negative for activated PKCε, whereas 30 min of stimulation with either PMA alone or uPA poorly induced its phosphorylation. However, PMA+uPA stimulation induced phosphorylation of PKCε in most cells (Figure 6B). Related to activated PKCδ PMA did not induce appreciable activation, whereas cell stimulation with either uPA alone or in the presence of PMA increased the number of cells positive for phosphorylated PKCδ (Figure 6B).

### Cytochalasin D (CytD) mimics the anti-HIV activities of uPA in PMA-stimulated U1 cells

We have previously reported that both β_1_ and β_2_ integrin chains mediate the antiviral effect of uPA in PMA-stimulated U1 cells as well as that integrin activation in the absence of uPA led to a similar effect [19]. Since integrin function is linked to the cytoskeleton activity we investigated the potential role of cytoskeletal elements in HIV expression and uPA-dependent effects. Therefore, U1 cells were stimulated in the presence or absence of CytD, a cell-permeable fungal toxin that prevents F actin polymerization and induces RhoA activation [32].

**Figure 7:**
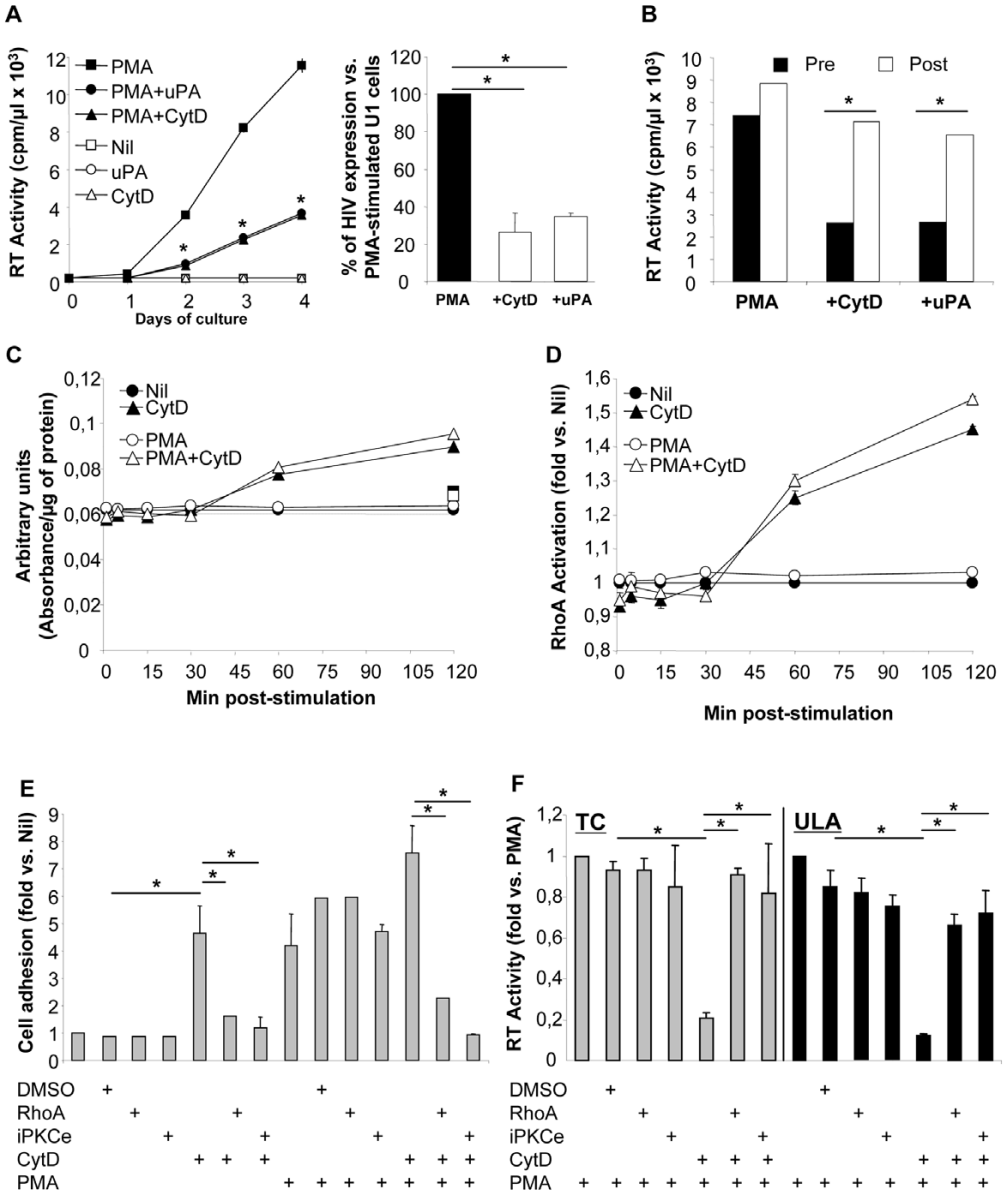
CytD inhibits late phase of HIV expression via activation of small GTPase RhoA and PKCε. (**A**) U1 cells were resuspended in culture medium supplemented of either CytD (10 µM) or 10 nM uPA and then left unstimulated or stimulated with PMA. Kinetics (left panel) and % of inhibition after 48 h of stimulation (right panel) are shown from 1 experiment representative of 4. (**B**) RT activity was measured after 72 h of stimulation in the supernatant of cells before and after being subjected to cycles of freezing and thawing. The results from 1 experiment out of 2 independently performed are shown. (**C**) RhoA activation in U1 cells upon CytD stimulation: absorbance indicating RhoA activation was measured by the G-LISA kit assay and was normalized per µg of protein. (**D**) Results of 3 independent experiments reported ad fold induction (mean ± STD) vs. unstimulated cells. (**E**) U1 cell adhesion was measured after 48 h of stimulation; the results are represented as fold induction vs. unstimulated U1 cells (Nil; with net OD values, subtracted of background, ranging between 0.05–0.06). (**F**) The RT activity was measured in the culture supernatants after 48 h of stimulation; the results are represented as fold of induction vs. PMA-stimulated U1 cells (with values ranging between 2,500–3,000 cpm/µl in standard plates (TC, tissue culture) allowing cell adhesion and 8,000–10,000 cpm/µl in ultra-low adhesion (ULA) plates, as reported [15]. Panels (**E**) and (**F**) show the average±SD of 4 independent experiments; control peptides, such as P1 and scrambled PKC pseudosubstrate produced results superimposable to those of DMSO. *p<0.05.

CytD did not influence the basal undetectable levels of virus expression in unstimulated U1 cells (Figure 7A), but it inhibited HIV release in PMA-stimulated cells to an extent comparable to that induced by uPA (Figure 7A). We next freeze-fractured CytD-treated cells and observed an increase in RT activity reaching levels to those of control PMA-stimulated cells, similar to what observed with uPA (Figure 7B), suggesting that the anti-HIV activity of CytD, as described for uPA [14], likely results in the intracellular budding and accumulation of virions; indeed, independent studies reported that CytD did not inhibit HIV proteins expression and maturation but HIV virion release in monocytic and T cell lines (U937 and Jurkat, respectively) [44], [45].

Consistently with independent findings [32], [46], we observed activated RhoA after 30 min of CytD incubation, an effect that lasted for at least 2 h, independently of the presence of PMA (Figure 7C). Similarly to what observed in uPA-stimulated cells (Figure 5A) the levels of RhoA activation were enhanced of about 50% (Figure 7D). Furthermore, CytD induced adhesion of unstimulated U1 cells, as reported for other cell types [47], and this effect was additive in the presence of PMA (Figure 7E).

**Figure 8:**
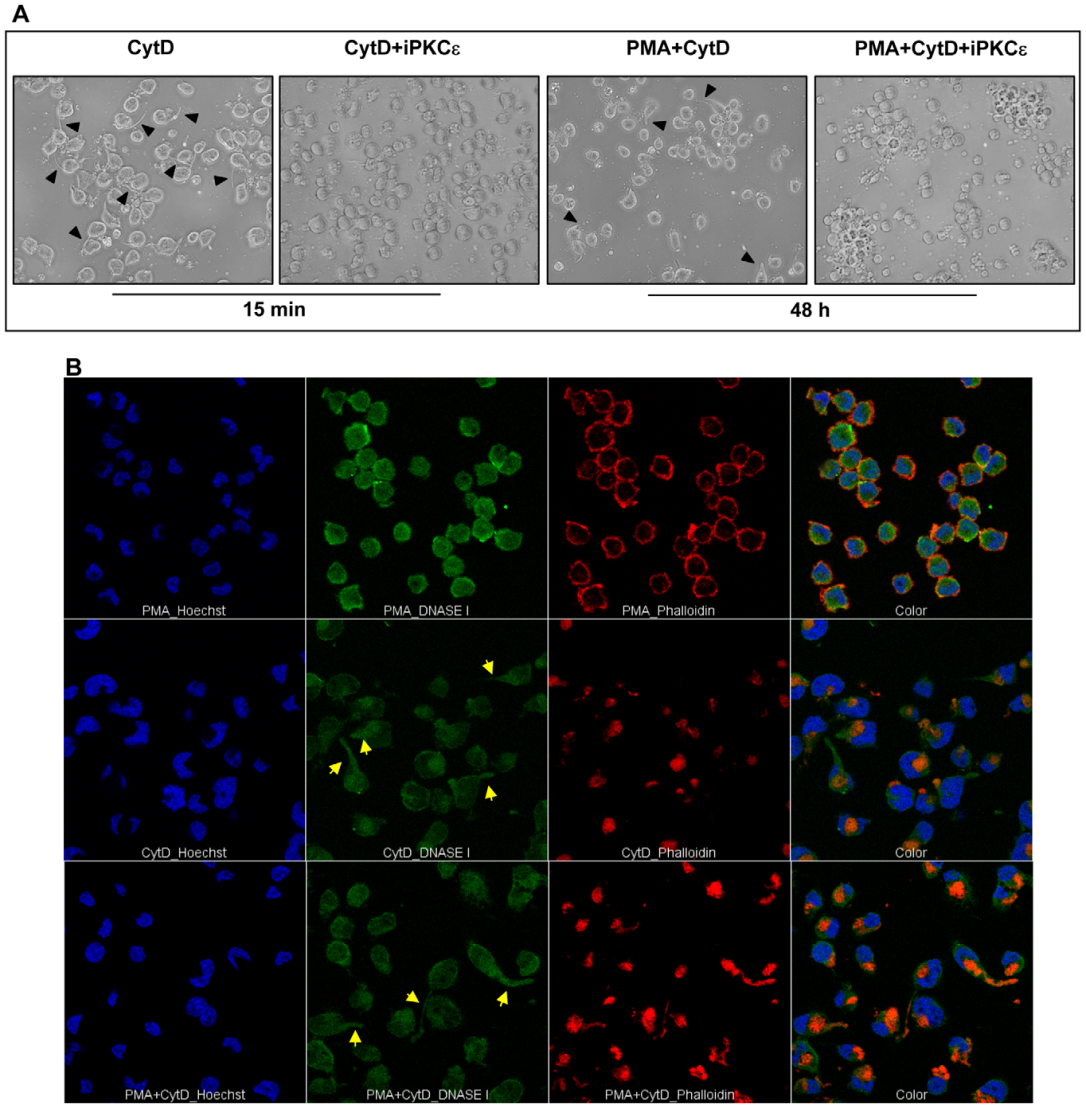
CytD induces U1 cell polarization and modification of actin distribution. U1 cells were stimulated with CytD in the presence or absence of PMA. (**A**) U1 cells were pre-incubated for 45–60 min at 37°C with the PKCε inhibitor and then stimulated with either CytD or CytD+PMA; cell polarization was visualized 15 min and 48 h after stimulation. Phase contrast pictures of cells were shot at 20× magnification; arrowheads indicate polarized structures. (**B**) G and F actin distribution in adherent U1 cells; upper panels show the effect of PMA stimulation, middle panels those of CytD stimulation and bottom panels of PMA+CytD co-stimulation. Staining of G and F actin, image acquisition and montage were performed as describe in the Figure 4 legend. Yellow arrows indicate polarized cells. One out of 3 independent experiments performed with similar results is shown.

CytD induced polarization of U1 cells, as reported on other cell types [47], [48], and also this effect was dependent upon PKCε activation (Figure 8A). CytD induced cell polarization as soon as after 15 min of stimulation and independently of cell adhesion. Moreover, like uPA, CytD also prevented the formation of cell aggregates in PMA-stimulated cells after 48 h of stimulation, another effect that was prevented by inhibition of PKCε activation (Figure 8A). We have also analyzed distribution of G and F actin in CytD adherent cells. As previously reported in fibroblasts [49], F actin resulted to be localized in focal aggregates (Figure 8B, middle panels), excluded from tails present in polarized cells. G actin was broadly distributed and also present in tail of polarized cells (Figure 8B, middle panels). PMA+CytD stimulated U1 cells were characterized by a morphology similar to that observed with CytD alone, although increased levels of intensity of F actin were noticed, probably due to the addictive adhesion effect induced by PMA and CytD (Figure 8B, bottom panels and Figure S6B).

As in U1 cells stimulated with PMA+uPA, CytD-induced cell adhesion was dependent on RhoA and PKCε activation (Figure 7E). Noteworthy, however, unlike uPA, the anti-HIV activity of CytD was not dependent on cell adhesion since superimposable results were obtained in Teflon-coated plates preventing cell adhesion (Figure 7F).

## Discussion

In the present study, we have investigated the signaling determinants involved in uPA-dependent inhibition of HIV-1 expression in a well defined model of macrophage infection such as chronically infected promonocytic U1 cells activated and differentiated by PMA. Indeed, several features typical of primary macrophage infection are observed in PMA-stimulated U1 cells, including the shift from growth in cell suspension to firm adhesion to the substrate coinciding with growth arrest [15] and the expression of the CD11b integrin chain [19], as also reported in the U1 uninfected parental cell line U937 [43]. Morphologically, PMA-differentiated U1 cells acquire a macrophage-like phenotype characterized by a ruffled plasma membrane and a vacuolarized cytoplasm where HIV frequently buds into and accumulate, particularly when IFN-γ or uPA are applied together with PMA [14], [23]. Finally, PMA-stimulated U1 cells resembles primary human MDM in terms of secretion of uPA, soluble uPAR (suPAR) and suPAR/uPA ratio [19].

Both the small GTPase RhoA and DAG-activable PKC isoforms δ and ε were activated by uPA. Along with increased U1 cell adhesion and inhibition of homotypic cell clustering, uPA induced polarization of PMA-stimulated U1 cells and RhoA activation. However, only RhoA and PKCε were linked to the intracellular accumulation of HIV virions, while uPA-dependent increased cell polarization and adhesion were dependent on RhoA, PKCδ and PKCε. CytD recapitulated through different dynamics all the essential features of uPA-dependent modulation, such as U1 cell adhesion, polarization and prevention of HIV virions release.

The uPA/uPAR complex has been involved in multiple biological processes and its dysregulation has been linked to important pathological conditions, including the formation of cancer metastasis and HIV/AIDS disease progression [3]. uPA activities can be subdivided in those processes consequent to its proteolytic component from those occurring after signaling mediated by uPAR association with a transducing receptor. The modulatory effects here investigated are strictly associated with this “second life” of uPA, such as cell adhesion, polarization and anti-HIV activity [14], [20]. uPA-induced chemotaxis of human vascular smooth muscle cells was reported to be mediated by activation of small GTPase RhoA and downstream phosphorylation of myosin light chain [50]. In this regard, the small GTPase RhoA has been previously implicated in chemoattractant-induced β_1_ [51] and β_2_ integrin mediated leukocyte adhesion, and, particularly, for α_L_β_2_ (LFA-1), in the regulation of heterodimer affinity increase and lateral mobility [30], [52], as well as in the enhancement of monocyte and promonocytic U937 cell adhesion to the substrate [53], [54] and very recently of neutrophil adhesion on fibrinogen [55].

Consistently, our previous [15] and current findings link them to the anti-HIV effect of uPA in various models of macrophage infection (including primary MDM and PMA-differentiated U1 cells) to an increased cell adhesion and to the activation of RhoA. Of interest is the fact that an inhibitory effect of transfected RhoA on HIV gene transcription and replication was demonstrated to occur in both adherent CD4-expressing 293T cells and in non-adherent Jurkat T lymphocytic cells [56]. In contrast, a positive role of RhoA activation has been described in dendritic cells following DC-SIGN activation and formation of an immunovirological synapse with T cells [57]. However, these two seemingly opposite effects may in fact represent two consequence of the same biological phenomenon: the concentration of virions in intracellular vesicles that could represent an inhibitory outcome for free virion release, but facilitate cell-cell virion transmission.

Downstream to RhoA [30], [33], activation of PKC is crucial for modulation of several cellular processes. In this regard, activation of PKC isoforms influences the morphology of the F-actin cytoskeleton and thereby regulates processes that are affected by remodeling of the microfilaments [42]. Integrins are crucial mediators both upstream and downstream of PKC in inducing morphological changes [42]. In addition, HIV virions and cytoskeleton elements strongly interact each other after the entry step or in order to bud from the infected cell as also evidenced by the incorporation of actin and actin-binding molecules into virions [58], [59]. Thus, PKC activation may lead to the regulation of virus expression either directly, by influencing viral transcription [60], [61] or by phosphorylating viral proteins [62], or indirectly via induction of cytoskeleton rearrangement. In particular, PKCε has been shown to phosphorylate vimentin (VM) [63] a cytoskeleton protein important for the cytoplasmic localization of the viral accessory protein Vif [64], therefore affecting virion infectivity [65]. VM activation can trigger the association of vesicle-bearing β_1_ integrins to intermediate filaments, thus allowing their movement towards the plasma membrane [63]. uPA-mediated activation of PKCε might regulate VM activity by altering its interactions with Vif and the viral protease [66] ultimately resulting in the accumulation of virions in intracellular vacuolar compartments. In this regard, blocking events downstream to PKCε erased the ability of uPA to induce intracellular vacuolization, a marker of functionally mature phagocytes [67]. Overall, these findings suggest that uPA-induced activation of PKCε is involved in pathways of host proteins stored into vesicles (i.e., needed for recycling of membrane receptors during chemotaxis), a system reported to be exploited by HIV in infected macrophages [68].

**Figure 9:**
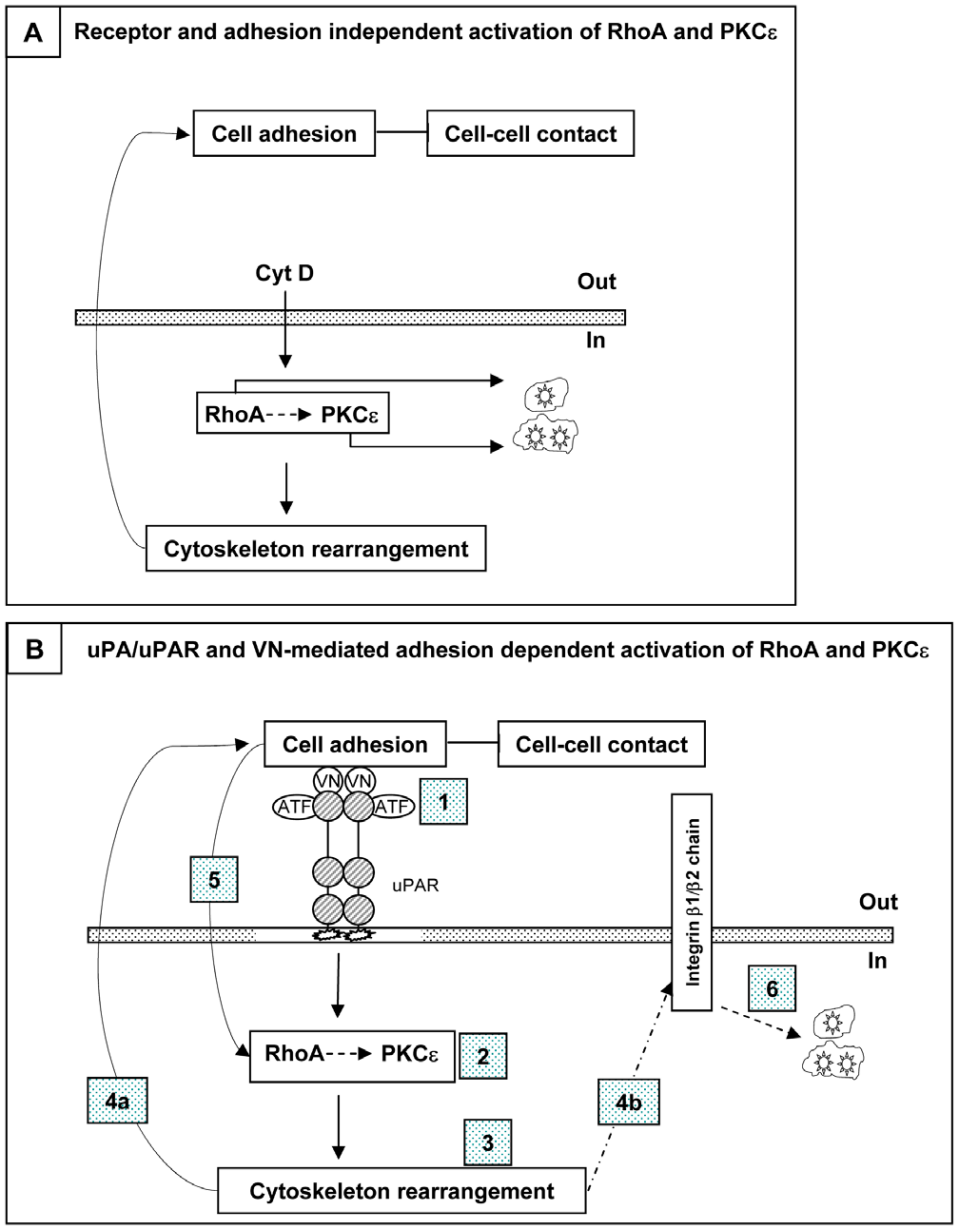
RhoA and PKCε activation leads to accumulation of HIV virions in cytoplasmic vacuolar compartments. (**A**) CytD mediated accumulation of virions is reversed by blocking RhoA and PKCε independently of cell adhesion. The same enzymes also modulate cytoskeletal rearrangement, as evident by cell polarization shown in Figure 8 and promote cell adhesion and inhibition of cell clustering [74]. (**B**) Binding of the signaling-competent portion of uPA (ATF) to uPAR recruits VN [15], [75] leading to dimerization of the complex likely occurring in lipid rafts [76] (white box inside the dashed box representing cell membrane, whereas stars represent GPI-anchor associated to uPAR) (**step 1**), independently of integrin activation [77], followed by early activation of RhoA and then PKCε (**step 2**). Cytoskeleton is rearranged (**step 3**) leading to two events, such as i) reinforcement of cell adhesion (**step 4a**) [32], [78] generating a second wave of RhoA activation (**step 5**), and ii) activation/clustering of β_1_ and β_2_ integrin chains (**step 4b**) [19] therefore generating a signaling cascade leading to the intracellular accumulation of virions in intracellular vesicles (**step 6**). Full arrows represent our experimental findings, whereas dashed arrows are hypothetical.

In order to better define the role of cytoskeleton in uPA-mediated activities, we also studied the effect of Cytochalasin D, a cell-permeable compound that does not require interaction with any membrane receptor to mediate both RhoA activation and cytoskeleton remodeling. CytD has been reported that it binds to the barber end of G actin thus preventing the following polymerization to F actin and leading to the accumulation of G actin. Moreover, it has been reported that F actin sequesters Rho guanine-nucleotide exchange factor, thus abrogating RhoA activation [69]; indeed, also microtubule disruption with either nocodazole or colchicines has been reported to increase global RhoA activity in neutrophils [70]. Consistently with this model we observed increased levels of activated RhoA upon 30 min of incubation, as reported independently [32], [46], after cytoskeletal rearrangement, an event that occurred as soon as after 15 min of treatment. Indeed, inhibition of RhoA reversed CytD-induced cell adhesion and inhibition of HIV release. Nonetheless, cell adhesion, polarization and inhibition of HIV release induced by CytD were also mediated by PKCε, an enzymatic activity reported to be downstream of RhoA-mediated signaling. Thus, a potential model of uPA/uPAR interaction may include the generation of an intracellular signal of reorganization of cytoskeleton components similar to that induced by CytD (Figure 9A) that inhibits virus replication at the level of virion assembly and release [45].

A potential working model connecting the multiple events induced by the uPA/uPAR system is shown in Figure 9B. According to this model, uPA/uPAR/VN complex likely triggers an outside-in signaling cascade facilitating cell adhesion and polarization of adherent cells. Since cell polarization occurred exclusively in adherent cells, but not in cells maintained in suspension in ULA plates, the intracellular signaling triggered by uPA, involving the activation of RhoA and PKC δ and ε, is crucial for the acquisition of cell polarity (i.e. formation of the uropods and, eventually, pseudopods), as previously discussed for epithelial cells [37], needed for migration on substrate. Conversely, the VN/uPA/uPAR complex might generate an “inside-out” signaling by perturbing the cytoskeleton organization triggering integrin activation that, in turn, may trigger an “outside-in” signaling pathway leading to cell polarization, as reported for CXCL12-dependent integrin activation, reported to induce cell adhesion and migration via activation of RhoA followed by activation of the integrin LFA-1 [41], [71], [72].

Thus, the uPA/uPAR/VN system favors macrophage adhesion and accumulation of virions in intracellular vesicles [68], highlighting a potential mechanism for the establishment and maintenance of this intra-cytoplasmic reservoir of preformed virions. Such a feature of virion morphogenesis has generated the model of macrophages as “Trojan horses” of infection, hiding infectious virions from the recognition of immune effectors such as neutralizing antibodies [68]. Finally, our results also extend uPA/uPAR-related findings to a broader model that involves pathways controlling cytoskeleton remodeling and activation of RhoA and PKCε as mediators of intracellular accumulation of HIV virions in vesicles as well as their release from infected macrophages.

The possibility of interfering with either the uPA/uPAR/VN system, RhoA or PKCε might therefore suggest novel targets to eliminate such an intracellular virion reservoir by facilitating virion release from tissue macrophages, including uPAR^+^/CD68^+^ macrophages accumulating virions described in the CNS of HIV^+^ individuals [13].

## Supporting Information

Figure S1 **UPA-mediated vesicles formation and accumulation of virions into vesicles are dependent of PKCε.** U1 cells were preincubated for 45–60 min at 37°C with myristoilated peptides specific for PKCε isoform and were then stimulated with PMA in the presence or absence of uPA for 48–72 h, then prepared for and analyzed by EM as described in material and methods. Four representative images are shown for each treatment (the third picture of the first panel shows enlargement of virion). Scale bar is reported at the bottom of each picture.
(TIF)

Figure S2 **PKCε does not influence IL-6 induced HIV expression.** U1 cells were preincubated for 45–60 min at 37°C with myristoilated peptides specific for PKCε isoform and were then stimulated IL-6, then prepared for and analyzed by EM as described in material and methods. Four representative images are shown for each treatment (the third picture of the first panel shows enlargement of virion). Scale bar is reported at the bottom of each picture. (**B**) Culture supernatants were analyzed 48 h later for the levels of virus expression (mean±SD of duplicate cultures).
(TIF)

Figure S3 **PMA-induced cell aggregation is prevented by uPA and reversed by blocking small GTPase RhoA and PKCδ or PKCεactivation.** (**A**) U1 cells were preincubated for 45–60 min at 37°C with either RhoA23 or RhoA92 Trojan peptides and were then stimulated with PMA in the presence or absence of uPA. Cells were left in their original culture well (not washed to allow persistence of both adherent and suspended cells). Pictures were shot 48 h later for visualizing homotypic cellular clustering (objective magnification 40×). The results of one experiments representative of 4 independently performed are shown. P1; penetratin was used as negative control of Trojan peptides. (**B**) U1 cells were pre-incubated for 45–60 min at 37°C with myristoylated peptides specific for different PKC isoforms and were then stimulated with PMA in the presence or absence of uPA. Cells were left in the original culture well and pictured 48 h later for homotypic cellular clustering (objective magnification 40×). The results of one experiments representative of 4 independently performed are shown. “Scramble” indicates an irrelevant myristoilated peptide used as negative control.
(TIF)

Figure S4 **uPA induced cell polarization is reversed by blocking RhoA and PKCε.** **A** and **B.** Cells were stimulated as described in Figure 1 and then washed to remove non-adherent cells. Arrowheads indicated polarized structures. Pictures (magnification: 40×) from 1 experiment representative of 11 independently performed are shown.
(TIF)

Figure S5 **Analysis of cell morphology and associated cell width/length ratio.** (**A**) U1 cells were loaded with cell tracker and then stimulated with PMA+uPA. Adherent cells were stained 2 days later for visualizing their nuclei. Representative cells (objective magnification of 40×) and their form factor are shown. Red dotted shapes are automatically generated by the IN Cell Investigator Software, based of the distribution of cell tracker, and used to calculate the form factor. (**B**) A total of 1142 and 2245 adherent cells were counted in PMA and PMA+uPA stimulated cells. Unlike what shown in Figure 4B, width/length ratio axis also includes non polarized cells, meaning ratios between 0.8 and 1. Panels **C** and **D** represent the absolute percentage of polarized (ratio below 0.8) and adherent cells and the inter-assay variation between experiments, respectively.
(TIF)

Figure S6 **Actin distribution in U1 stimulated cells.** U1 cells were stimulated with PMA in the presence or absence of uPA/CytD and actin distribution in adherent cells was visualized after 48 h of culture by confocal microscopy. Hoechst-33342, DNase I (Alexa Fluor 488, green) and phalloidin (Alexa Fluor 633, red) were used to discriminate nuclei, globular (G actin) and filamentous (F actin) isoforms, respectively. Upper panels show PMA stimulated U1 cells in abscence or presence of uPA; bottom panels show CytD stimulated U1 cells in absence or presence of PMA. Image J software was used to perform montage of the three colors. One experiment out of three with similar results is shown. Images were acquired with 63× magnification by Leica TCS SP2 confocal microscope.
(TIF)

Figure S7 **Different modulation of G and F actin distribution by uPA and CytD in stimulated U1 cells.** U1 cells were stimulated and treated as described in the legend of figure S5. Upper panels show G-actin distribution in U1 cells cultivated with all different stimuli; bottom panels show F-actin in the same stimulated U1 cells. Images were acquired with 63× magnification by Leica TCS SP2 confocal microscope and electronically zoomed 3 times.
(TIF)
