# Supplementary figures and images for: Urokinase Plasminogen Activator Inhibits HIV Virion Release from Macrophage-Differentiated Chronically Infected Cells via Activation of RhoA and PKCε
Source: PLoS One. 2011 Aug 17;6(8):e23674. doi: 10.1371/journal.pone.0023674 (PMC3157461; doi:10.1371/journal.pone.0023674)

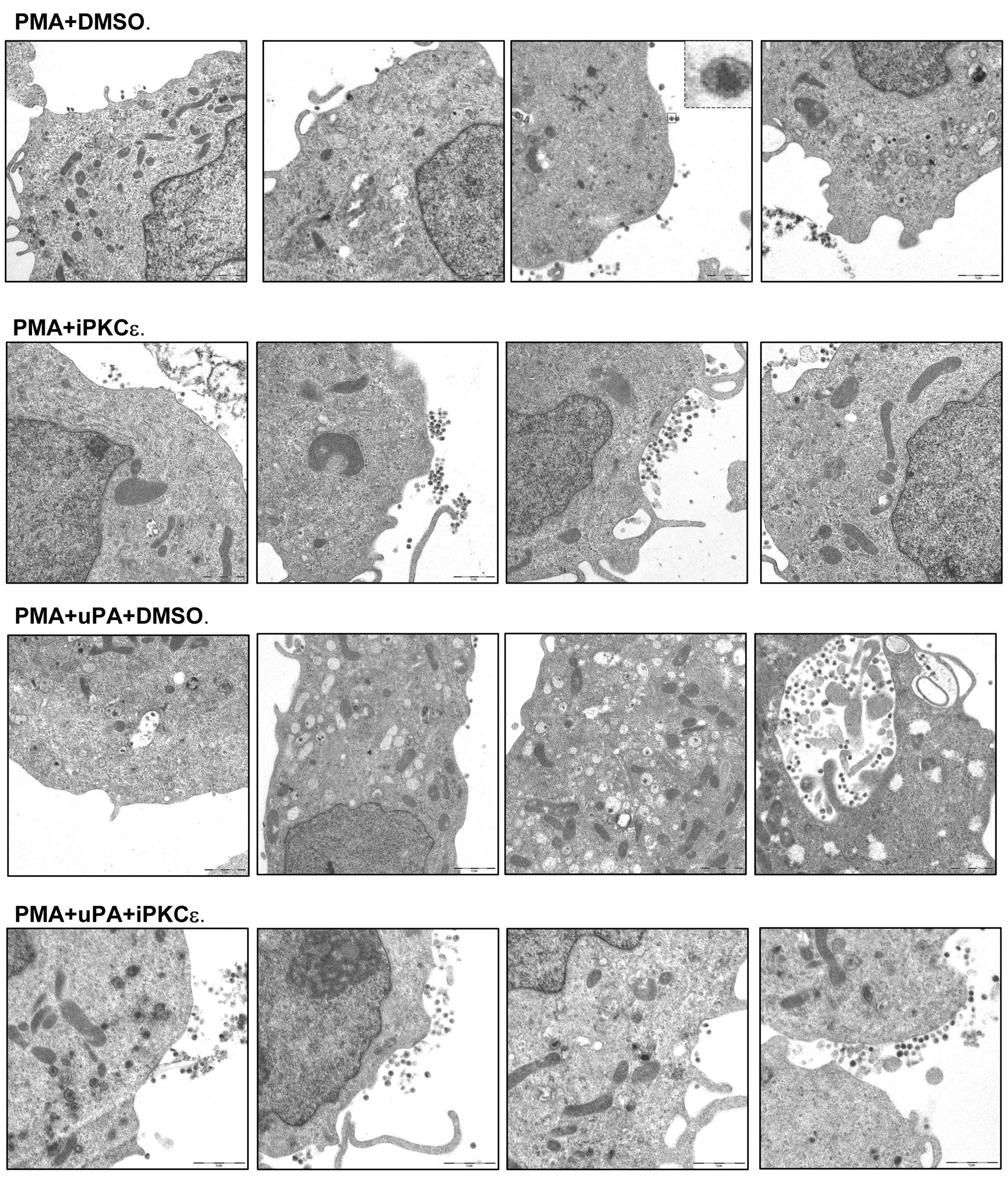

Supplement: Figure S1 — UPA-mediated vesicles formation and accumulation of virions into vesicles are dependent of PKCε. U1 cells were preincubated for 45–60 min at 37°C with myristoilated peptides specific for PKCε isoform and were then stimulated with PMA in the presence or absence of uPA for 48–72 h, then prepared for and analyzed by EM as described in material and methods. Four representative images are shown for each treatment (the third picture of the first panel shows enlargement of virion). Scale bar is reported at the bottom of each picture. (TIF) [file pone.0023674.s001.tif]

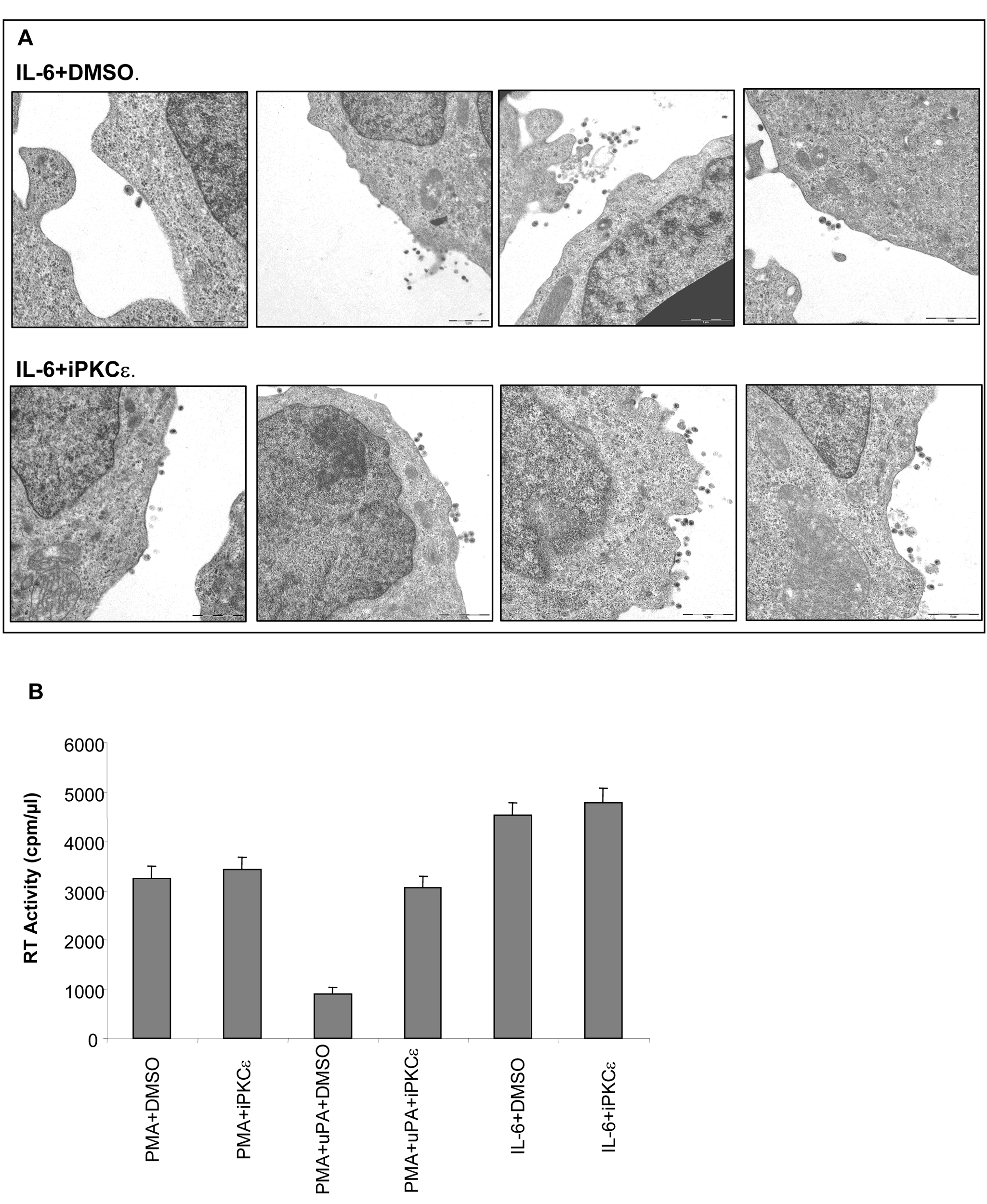

Supplement: Figure S2 — PKCε does not influence IL-6 induced HIV expression. U1 cells were preincubated for 45–60 min at 37°C with myristoilated peptides specific for PKCε isoform and were then stimulated IL-6, then prepared for and analyzed by EM as described in material and methods. Four representative images are shown for each treatment (the third picture of the first panel shows enlargement of virion). Scale bar is reported at the bottom of each picture. (B) Culture supernatants were analyzed 48 h later for the levels of virus expression (mean±SD of duplicate cultures). (TIF) [file pone.0023674.s002.tif]

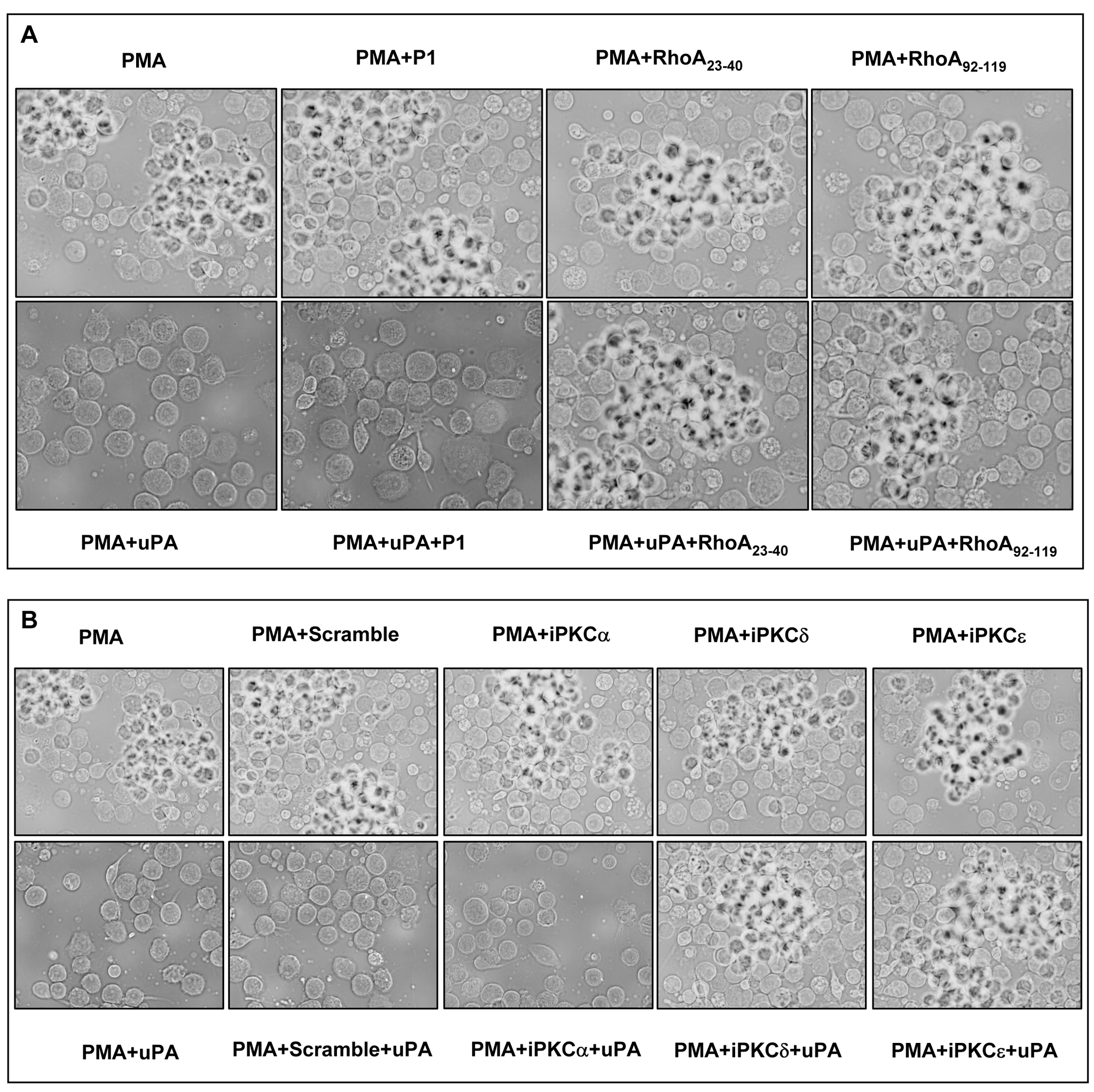

Supplement: Figure S3 — PMA-induced cell aggregation is prevented by uPA and reversed by blocking small GTPase RhoA and PKCδ or PKCεactivation. (A) U1 cells were preincubated for 45–60 min at 37°C with either RhoA23 or RhoA92 Trojan peptides and were then stimulated with PMA in the presence or absence of uPA. Cells were left in their original culture well (not washed to allow persistence of both adherent and suspended cells). Pictures were shot 48 h later for visualizing homotypic cellular clustering (objective magnification 40×). The results of one experiments representative of 4 independently performed are shown. P1; penetratin was used as negative control of Trojan peptides. (B) U1 cells were pre-incubated for 45–60 min at 37°C with myristoylated peptides specific for different PKC isoforms and were then stimulated with PMA in the presence or absence of uPA. Cells were left in the original culture well and pictured 48 h later for homotypic cellular clustering (objective magnification 40×). The results of one experiments representative of 4 independently performed are shown. “Scramble” indicates an irrelevant myristoilated peptide used as negative control. (TIF) [file pone.0023674.s003.tif]

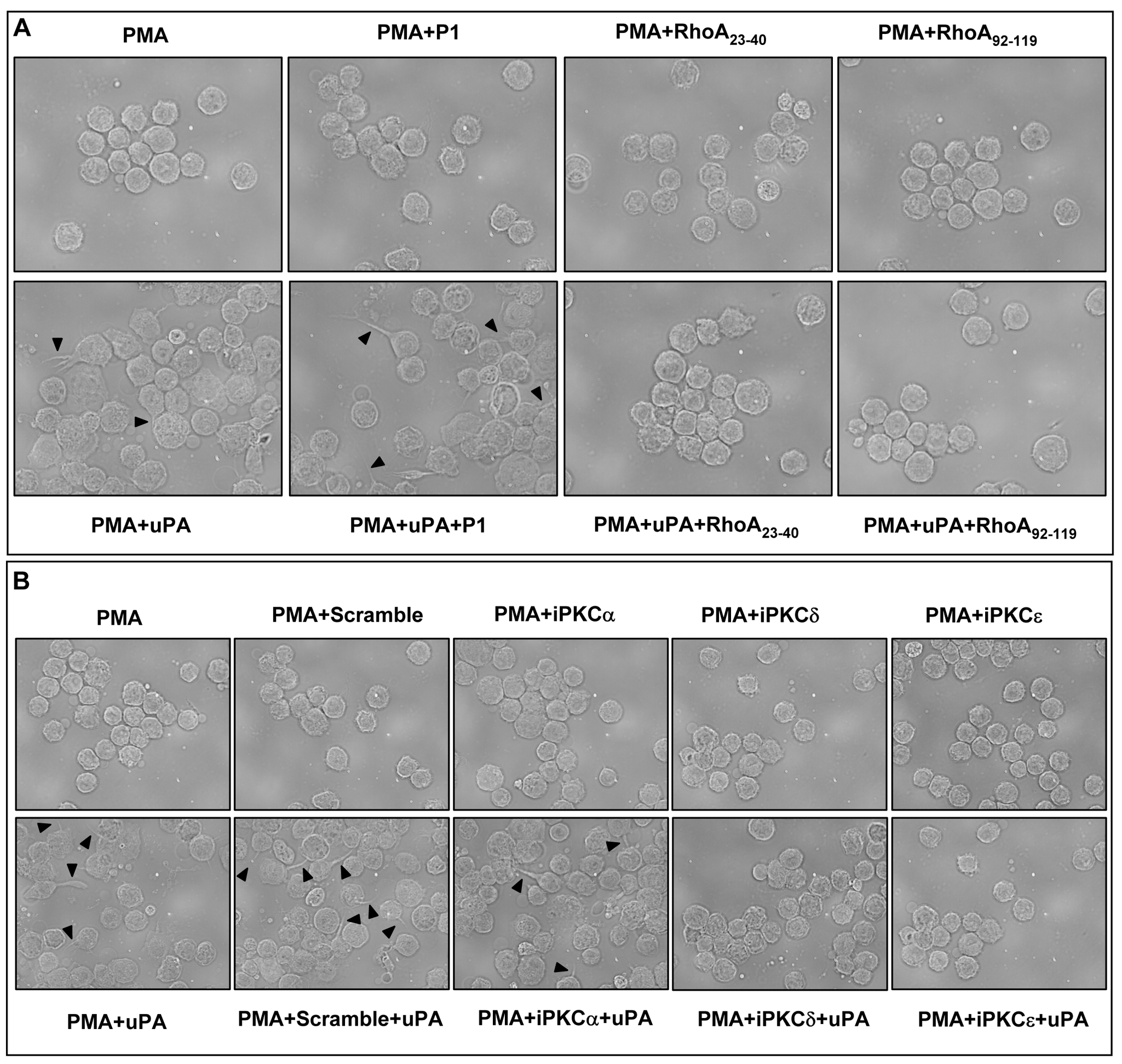

Supplement: Figure S4 — uPA induced cell polarization is reversed by blocking RhoA and PKCε. A and B. Cells were stimulated as described in Figure 1 and then washed to remove non-adherent cells. Arrowheads indicated polarized structures. Pictures (magnification: 40×) from 1 experiment representative of 11 independently performed are shown. (TIF) [file pone.0023674.s004.tif]

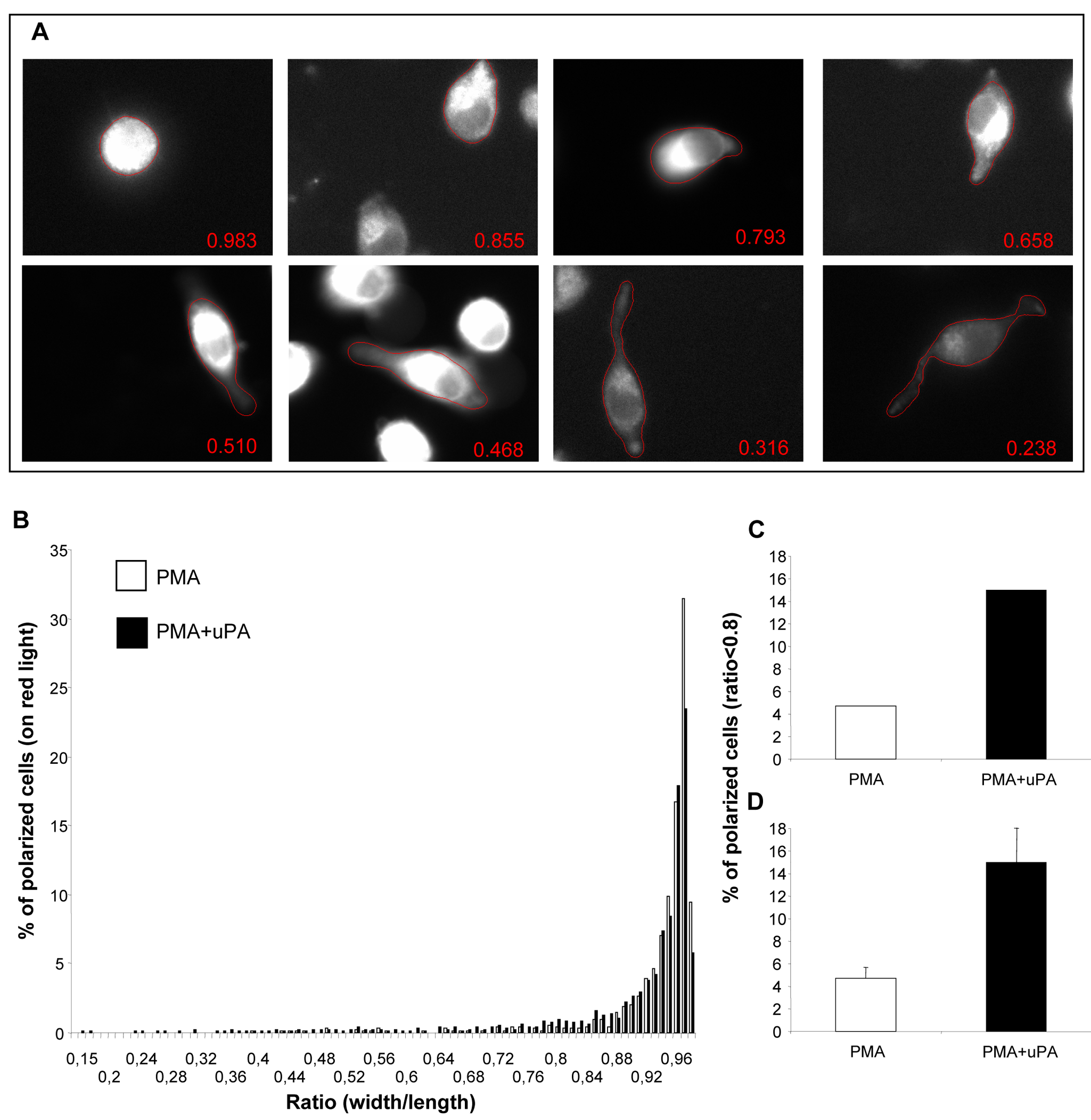

Supplement: Figure S5 — Analysis of cell morphology and associated cell width/length ratio. (A) U1 cells were loaded with cell tracker and then stimulated with PMA+uPA. Adherent cells were stained 2 days later for visualizing their nuclei. Representative cells (objective magnification of 40×) and their form factor are shown. Red dotted shapes are automatically generated by the IN Cell Investigator Software, based of the distribution of cell tracker, and used to calculate the form factor. (B) A total of 1142 and 2245 adherent cells were counted in PMA and PMA+uPA stimulated cells. Unlike what shown in Figure 4B, width/length ratio axis also includes non polarized cells, meaning ratios between 0.8 and 1. Panels C and D represent the absolute percentage of polarized (ratio below 0.8) and adherent cells and the inter-assay variation between experiments, respectively. (TIF) [file pone.0023674.s005.tif]

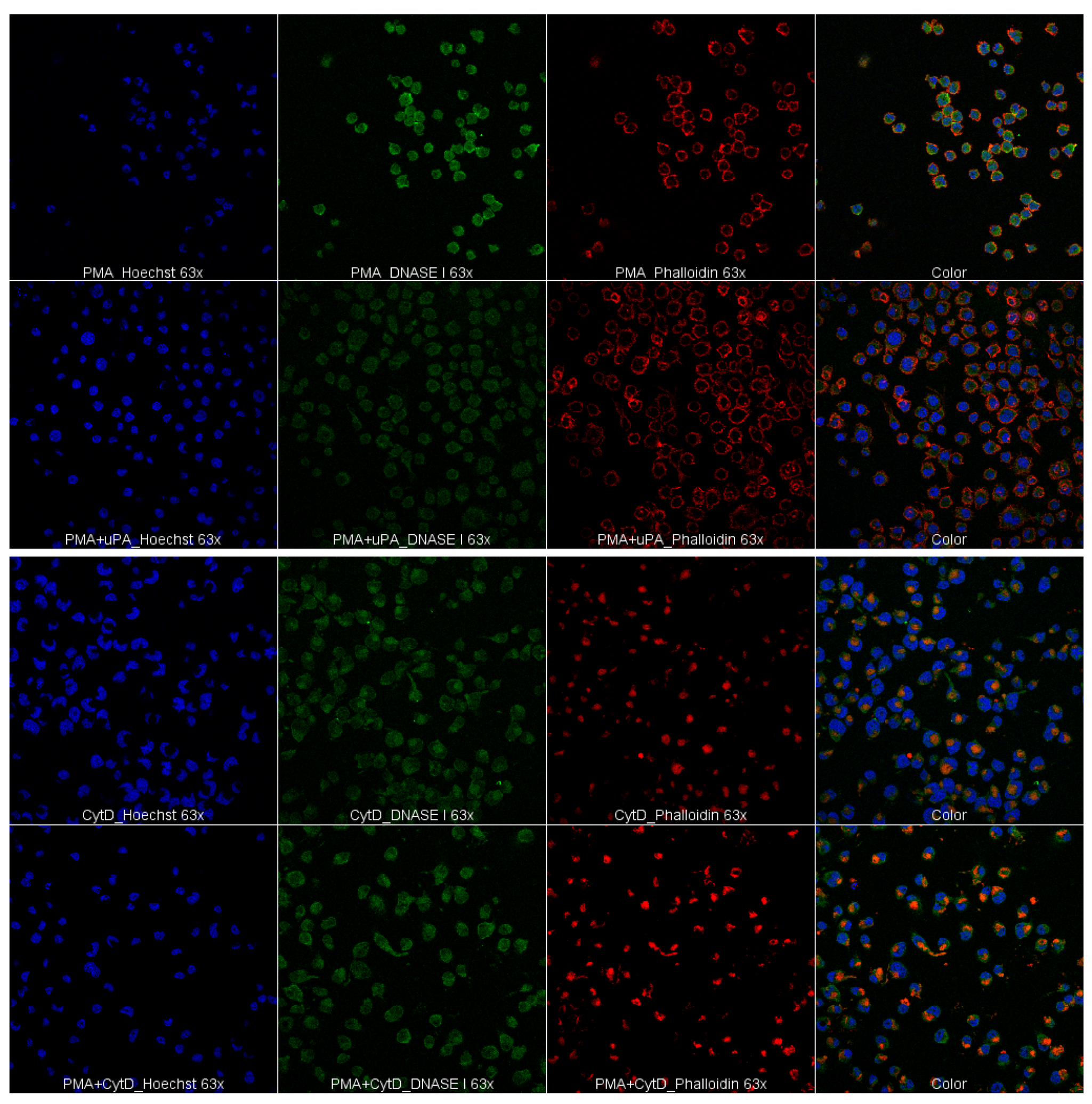

Supplement: Figure S6 — Actin distribution in U1 stimulated cells. U1 cells were stimulated with PMA in the presence or absence of uPA/CytD and actin distribution in adherent cells was visualized after 48 h of culture by confocal microscopy. Hoechst-33342, DNase I (Alexa Fluor 488, green) and phalloidin (Alexa Fluor 633, red) were used to discriminate nuclei, globular (G actin) and filamentous (F actin) isoforms, respectively. Upper panels show PMA stimulated U1 cells in abscence or presence of uPA; bottom panels show CytD stimulated U1 cells in absence or presence of PMA. Image J software was used to perform montage of the three colors. One experiment out of three with similar results is shown. Images were acquired with 63× magnification by Leica TCS SP2 confocal microscope. (TIF) [file pone.0023674.s006.tif]

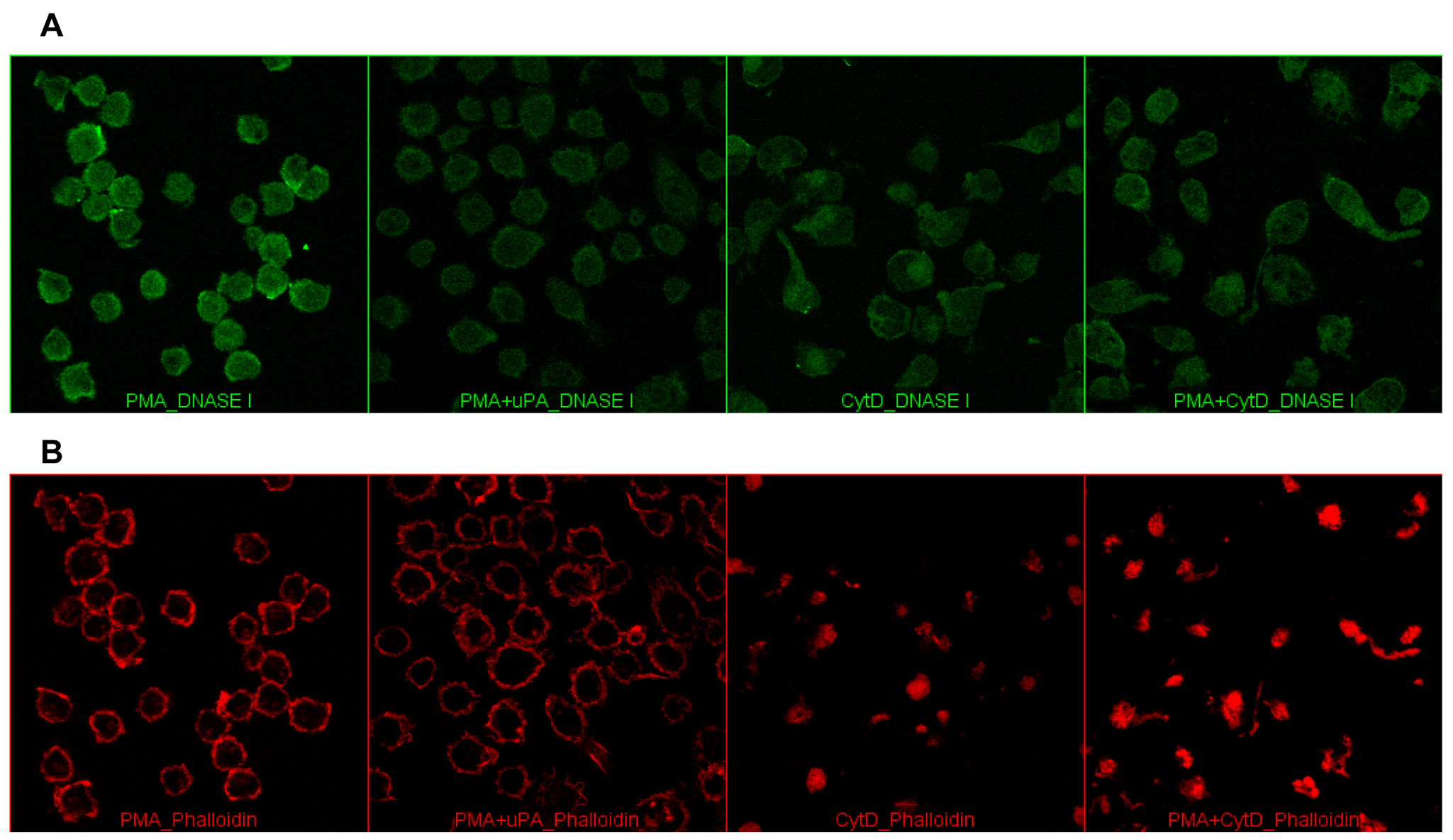

Supplement: Figure S7 — Different modulation of G and F actin distribution by uPA and CytD in stimulated U1 cells. U1 cells were stimulated and treated as described in the legend of figure S5. Upper panels show G-actin distribution in U1 cells cultivated with all different stimuli; bottom panels show F-actin in the same stimulated U1 cells. Images were acquired with 63× magnification by Leica TCS SP2 confocal microscope and electronically zoomed 3 times. (TIF) [file pone.0023674.s007.tif]
